# Evidence for topological type-II Weyl semimetal WTe_2_

**DOI:** 10.1038/s41467-017-02237-1

**Published:** 2017-12-15

**Authors:** Peng Li, Yan Wen, Xin He, Qiang Zhang, Chuan Xia, Zhi-Ming Yu, Shengyuan A. Yang, Zhiyong Zhu, Husam N. Alshareef, Xi-Xiang Zhang

**Affiliations:** 1 King Abdullah University of Science and Technology (KAUST), Physical Science and Engineering Division (PSE), Thuwal, 23955-6900 Saudi Arabia; 2 Research Laboratory for Quantum Materials, Singapore University of Technology and Design, Singapore, 487372 Singapore; 3 King Abdullah University of Science and Technology (KAUST), KAUST Supercomputing Laboratory (KSL), Thuwal, 23955-6900 Saudi Arabia

## Abstract

Recently, a type-II Weyl fermion was theoretically predicted to appear at the contact of electron and hole Fermi surface pockets. A distinguishing feature of the surfaces of type-II Weyl semimetals is the existence of topological surface states, so-called Fermi arcs. Although WTe_2_ was the first material suggested as a type-II Weyl semimetal, the direct observation of its tilting Weyl cone and Fermi arc has not yet been successful. Here, we show strong evidence that WTe_2_ is a type-II Weyl semimetal by observing two unique transport properties simultaneously in one WTe_2_ nanoribbon. The negative magnetoresistance induced by a chiral anomaly is quite anisotropic in WTe_2_ nanoribbons, which is present in *b*-axis ribbon, but is absent in *a*-axis ribbon. An extra-quantum oscillation, arising from a Weyl orbit formed by the Fermi arc and bulk Landau levels, displays a two dimensional feature and decays as the thickness increases in WTe_2_ nanoribbon.

## Abstract (web summary)

Exotic transport properties of type-II Weyl semimetals have been predicted but are yet to be experimentally evidenced. Here, Li et al. report evidences of an anisotropy of negative magnetoresistance and a quantum oscillation arising from the predicted Weyl orbit in the type-II Weyl semimetal WTe_2_.

## Introduction

The three-dimensional (3D) topological semimetals, Dirac and Weyl, have been extensively investigated as representatives of a new state of topological quantum matter that, in the bulk state, possesses electrons with linear dispersion at the Dirac or Weyl points^1–17^. Owing to the unique properties of these electrons in the bulk state (e.g., massless and defined chirality), other exotic physical properties have also been discovered, e.g., an unsaturated, positive magnetoresistance (MR) and an ultrahigh mobility of the carriers^5,18^. In addition to having unique bulk state electrons and novel topological surface states, the so-called Fermi arcs also exist on these surfaces with broken inversion symmetry^6^. A new quantum oscillation frequency originating from these Fermi arcs, observed in the Dirac semimetal Cd_3_As_2,_ is also predicted to exist in ultrathin slabs of topological Dirac and Weyl semimetals^12,19^.

**Fig. 1:**
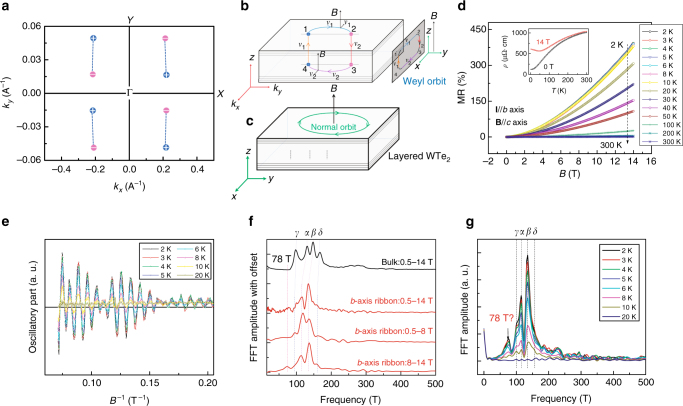
Fermi-arc-induced Weyl orbit oscillations. **a** The calculated Weyl points and a possible Fermi arc in the *k*_*z*_ = 0 plane of WTe_2_^6,13^. The Fermi arcs are mainly along Y direction. **b** Schematic of a Fermi-arc-induced Weyl orbit in a thin WTe_2_ nanoribbon, in which the magnetic field is along the *z*-axis (or *c*-) axis. This schematic is similar to that proposed by Potter et al.^19^. This Weyl orbit is formed by connecting two bulk paths along the *z*-direction through the zeroth chiral bulk Landau level (LL) and two Fermi arcs in the momentum space, on both the top and bottom surfaces. The trajectory of the Weyl orbit in real space is in the *xz* plane, and the Weyl orbit is plotted in a combination of real space and momentum space. The Weyl points with opposite chirality are labeled as + (blue) and – (purple). **c** Conventional quantum oscillation orbit. **d** MR of the *b*-axis ribbon (19.4 nm thick) at different temperatures with **B**//*c*. Inset shows a metal–insulator transition under a magnetic field of 14 T. **e** The pronounced SdH oscillations are observed at different temperatures in the plots of d^2^ *R*/d*B* ^2^ vs. *B*. **f** Comparison of FFT of the data of d^2^ *R*/d*B* ^2^ vs. *B* obtained from bulk WTe_2_ and *b*-axis ribbon (*T = *2 K). The FFT data obtained from d^2^ *R*/d*B* ^2^ vs. *B* for *B* > 8.0 T shows an extra frequency of 78 T. **g** The FFT spectra of *b*-axis ribbon at different temperatures

WTe_2_ was suggested as the first material candidate for a type-II Weyl semimetal, in which the Weyl points occur at the crossing of the oblique conduction and the valance bands (Weyl cone tilts along *Y* axis in momentum space, Fig. 1a)^6^. It was also predicted that there exist eight separated Weyl points in the bulk of WTe_2_ and Fermi arcs on the (001) crystal surfaces owing to reflection symmetry. These Weyl points with opposite chiralities are possibly connected by Fermi arcs with a separation of 0.032 Α^−1^ along *Y* axis in momentum space (Fig. 1a)^13,14^. According to the definition of a type-II Weyl semimetal, a quasiparticle can be regarded as a type-II Weyl fermion that has the following characteristics. Along the *k* direction that Weyl cone tilts toward, its kinetic energy is larger than its potential one in low-energy condensed matter physics^6^. More specially, type-II Weyl fermions violate the Lorentz symmetry strongly^6,20^. Recently, it was reported that the decisive criterion for a type-II Weyl semimetal is the direct observation of its tilted band crossing in three directions in the momentum space, as demonstrated in LaAlGe^20^. However, experimental demonstrations of type-II Weyl fermions in WTe_2_ have been unsuccessful owing to the limited resolution of angle-resolved photoemission spectroscopy (ARPES) and scanning tunneling spectroscopy, which prevents direct observation of the tilting Weyl cone and Fermi arcs in WTe_2_^8,13,14,21^.

It has been established that the anisotropy of chiral anomaly should be a strong evidence to distinguish the type-II Weyl semimetals from type-I Weyl semimetals, in addition of the direct observation of the tilted bulk Weyl cones using ARPES^6,22^. Chiral anomaly refers to the state at which the particle number with given chirality is non-conserved. A negative MR caused by this chiral anomaly should be observed when a magnetic field is applied parallel to the electric field^23^. In type-I Weyl semimetals, the chiral anomaly always appears regardless of the direction of the applied magnetic fields. However, in type-II Weyl semimetals, the chiral Landau levels and chiral anomaly exists only when the magnetic field is applied in the direction along which the kinetic energy of the fermions is larger than their potential energy^6,22,24–26^. Specifically, the ratio of the kinetic energy (*T*_k_) to potential energy (*U*_k_), *R* = *T* ^2^(k)/*U* ^2^(k) > 1 was proposed to be a criterion to identify the directions along which a Weyl fermion is type-II and has the chiral anomaly^6,22^. This phenomenon should be a direct evidence for type-II Weyl semimetals. Moreover, a new cyclotron motion in Weyl and Dirac semimetals, the Weyl orbit, is proposed to be formed by connecting these surface Fermi arcs via the Weyl points in the bulk crystal^19^. This Weyl orbit will lead to an additional frequency in the magnetic quantum oscillation spectra for experiments that probe the Fermi surfaces, such as magneto-transport measurements. The observation of the extra Weyl orbit frequency is indirect evidence, because it cannot provide the information about the type of a Weyl semimetal. It is important to note that the Weyl orbit in type-II Weyl semimetals will be slightly modified due to the absence of chiral Landau levels^24–26^, when the magnetic field is normal to the tilt of Weyl cone. However, the main conclusions keep the same as in type-I Weyl semimetals (Supplementary Note 1). Therefore, we are motivated to explore these two exotic properties, the evidence for type-II Weyl semimetal WTe_2_, through magneto-transport measurements.

In this work, we use electrical transport measurements to demonstrate that WTe_2_ is a type-II Weyl semimetal with topological Fermi arcs. Based on the theoretical prediction and previous experimental results, a material can be viewed as a type-II Weyl semimetal if we observe extra-quantum oscillation due to the Weyl orbit formed by a connection between Fermi arcs and bulk Weyl points^12,19^, and an anisotropic negative MR caused by a chiral anomaly^6,22,24–26^. Below, we show that these two characteristics of a type-II Weyl semimetal are clearly observed simultaneously on the same piece of WTe_2_ nanoribbons.

## Results

### Extra-quantum oscillation from Weyl orbit

According to the theoretical prediction, topological surface states exist only on the (001) planes of WTe_2_^6^. To study the transport anisotropy, we patterned nanoribbons from the longest *a*-axis and *b*-axis sides of the same slab of exfoliated WTe_2_ (*a*-axis and *b*-axis ribbon, Supplementary Fig. 1). To avoid complexity caused by the extrinsic effect, such as severe scattering of the ribbon boundaries, all of the ribbons were fabricated with a width larger than 0.6 μm (Supplementary Fig. 2). Moreover, as the thickness of the WTe_2_ ribbons decreased to 5.6 nm, the electrical transport behavior transformed gradually from metallic to insulating; particularly, the size of MR was largely suppressed (Supplementary Fig. 3), which is consistent with previous work^27^. Therefore, a *b*-axis ribbon with a moderate thickness *t = *19.4 nm (~14 atomic layers) was chosen for a detailed investigation of its magneto-transport properties (Supplementary Fig. 4a). The observations of a significantly large MR (>400%) for both **I**//*b* and **B**//*c* at low temperatures, and of a metal–insulator transition under the high magnetic field *B* = 14 T (Fig. 1d and its inset), indicate clearly that the 19.4 nm thick ribbon still remains in the bulk state of WTe_2_^18^. Below 20 K (Fig. 1e), we also observe pronounced Shubnikov–de Hass (SdH) oscillations from the second-order derivatives, d^2^ *R*/d*B* ^2^, of the data in Fig. 1d, indicating the high quality of the nanoribbons.

To understand the data obtained from the *b*-axis nanoribbon (Fig. 1e), we carried out the same measurements on bulk single crystals with the same configuration (**I**//*b* and **B**//*c*). The fast Fourier transformation (FFT) of the second-order derivative of the MR obtained from both the nanoribbon and the bulk sample are compared in Fig. 1f. Clearly, the frequencies of the bulk crystal agree well with previous reports that the quantum oscillations originate from four ellipsoidal Fermi surfaces, two electron pockets (*α* and *β*) and two hole pockets (*γ* and *δ*)^28,29^. Compared to the frequencies of the bulk crystal, the *b*-axis ribbon data show three interesting features: decreasing/vanishing of the peaks of the two hole pockets (*γ* and *δ*); the positions of the two electron pocket peaks (*α* and *β*) shift much more than the peaks of the two hole pockets (*γ* and *δ*); and the appearance of an extra peak at 78.0 T. The diminishing of the peaks at the two hole pockets indicate that the electronic transport of the ribbon is dominated by electrons, which may suggest a rise in the Fermi level of the ribbon compared to its bulk counterpart. Notably, the predicted Weyl points are about 50–80 meV above the Fermi level^6^; therefore, a higher Fermi level in the nanoribbon may make it possible for us to detect the potential Weyl points through the transport measurements. Compared to the FFT data of bulk WTe_2_, frequencies in nanoribbons correspond to different Fermi surface sizes, i.e., shift in the chemical potential. The shift of peak positions is probably caused by the Fermi triaxial ellipsoids shrinking in volume as the ribbon thickness decreases and Fermi level increases^30^, which is also supported by the gradual opening of the bandgap as the thickness of WTe_2_ decreases (Supplementary Fig. 3). To explore the origin of the peak at 78.0 T, we analyze the data more carefully, finding that this extra peak came from the data collected under a magnetic field higher than 8.0 T, and that only four peaks corresponding to bulk Fermi surfaces can be observed below 8.0 T. Figure 1g shows the FFT data from Fig. 1d at different temperatures. The strength of the peaks decreases quickly as the temperature increases. This temperature dependence can be well-fitted by the Lifshitz–Kosevich equation, which leads to effective masses of 0.412*m*_0_ ± 0.021*m*_0_(*α*), 0.482*m*_0_ ± 0.024*m*_0_(*β*), and 0.253*m*_0_ ± 0.013*m*_0_ (78.0 T) (Supplementary Fig. 4b–d). The effective masses of *α* and *β* in the ribbon agree well with those in bulk WTe_2_^28^.

To understand the physics of the extra peak at 78.0 T, let us first examine whether the semi-classical Weyl orbit model^19^ can be applied here. In this model, a cyclotron motion formed by the chiral Landau levels and the Fermi arcs may result in an extra-quantum oscillation in Dirac or Weyl semimetals; this was successfully applied to interpret the experimental observation, particularly to demonstrate the existence of the non-trivial surface states on a slab of the Dirac semimetal, Cd_3_As_2_^12^. Nevertheless, several calculations suggest that chiral Landau levels in type-II Weyl semimetals will be absent when the applied magnetic field (**B**//*z*) is normal to the tilting direction of Weyl cone (*Y* direction^24–26^. Therefore, the semi-classical Weyl orbit in type-II Weyl semimetals should be slightly modified. To form a Weyl orbit, the bulk Landau levels will play the role of the chiral Landau levels to connect the Fermi arcs as in the type-I and Dirac semimetals. This modification in the model of Weyl orbit will not change the main conclusions (see Supplementary Note 1 in details). If the electric field is applied along the *k*_*y*_ (*b*) direction and the magnetic field is applied along the *z* (or *c*) direction in the *b*-axis ribbon of WTe_2_, a complete Weyl orbit will form (Fig. 1b) and correspond to a trajectory in the *xz* plane in real space. A unique feature of the Weyl orbit is that the magnetic field is parallel to the orbit in real space, rather than normal to the orbits of the conventional quantum oscillations (Fig. 1c). According to this model, the frequency of the quantum oscillation due to this Weyl orbit can be estimated by ref. ^19^

1 $$F_{\mathrm{S}} = \frac{{E_{\mathrm{F}}k_0}}{{e\pi \bar v_{\mathrm{F}}}},$$

where $E_{\mathrm{F}} = \hbar \bar v_{\mathrm{F}}\bar k_c$ and $\bar v_{\mathrm{F}} = \hbar \bar k_c/\bar m^ *$are the bulk Fermi energy and Fermi velocity, respectively, of electrons sliding along the *c* direction via the chiral bulk LLs; *k*_0_ is Fermi-arc separation.

Therefore, to obtain the frequency *F*_S_ of the Weyl orbit in a *b*-axis ribbon, we extract *E*_F_, *v*_F_, and the Fermi radius *k*_*c*_ from the SdH oscillations obtained from the bulk WTe_2_. The Fermi radius can be calculated using the Onsager equation *F* = ($\hbar /2{\mathrm{\pi e}}$)*S*_F_^31^, where *S*_F_ is the extreme cross-sectional area of the 3D Fermi surface. All four of the Fermi surface pockets mapped by quantum oscillations are triaxial ellipsoidal rather than spherical (Supplementary Fig. 5 and Supplementary Table 1). Since the electronic transport in the WTe_2_ ribbons is dominated by electrons, we only need the values of *k*_F_ for the two electron pockets along the *c* direction (*α* and *β* in Fig. 1f). The radii of the two electron pockets are obtained as *k*_*αc*_ = 1.19 nm^−1^ and *k*_*βc*_ = 1.22 nm^−1^ (Supplementary Table 1). These values are in good agreement with previous quantum analyses from Seebeck measurements^28^. The Fermi velocity of bulk electrons in a Weyl orbit and the bulk Fermi energy are obtained as

2 $$\bar v_{\mathrm{F}} = \frac{{\hbar \bar k_c}}{{\bar m^ * }} \approx 3.09 \times 10^5\,{\mathrm{ms}}^{{\mathrm{ - 1}}},$$

3 $$E_{\mathrm{F}} = \hbar \bar v_{\mathrm{F}}\bar k_c \approx 244.5\,{\mathrm{meV}}{\mathrm{.}}$$

Thus, we calculate the frequency of the quantum oscillation caused by this Weyl orbit using Eq. (1) as *F*_S_ ≈ 80.6 T ± 4.0 T, where the value of the Fermi-arc separation *k*_0_ is 0.032 Α^−1^ ± 0.002 Α^−1^ ^13^. This value agrees well with the experimental observation of 78.0 T ± 3.9 T. And two more sets of data with Weyl orbit frequency and effective mass analysis are shown in Supplementary Figs. 6 and 7. The observation of the coherent quantum oscillation associated with the Fermi arc requires that *ω*_c_ *τ* = *ev*_F_ *Bτ*/2*k*_0_ > 1^19^, i.e., the electrons must finish a complete Weyl orbit (Fig. 1b) before being scattered off. For a quantum scattering time $\bar \tau = \left( {1.6 \pm {\mathrm{0}}{\mathrm{.1}}} \right) \times 10^{ - 13}{\mathrm{s}}$ (Supplementary Fig. 8), the quantum oscillations associated with Fermi arcs can be observed only when the applied magnetic field *B* > 8.6 T, which is consistent with the observation of a 78.0 T peak only when *B* > 8.0 T (Fig. 1f, e). Thus, we conclude that we have indeed demonstrated the existence of a Fermi-arc-induced quantum oscillation in WTe_2_.

### Two-dimensional feature of Weyl orbit

**Fig. 2:**
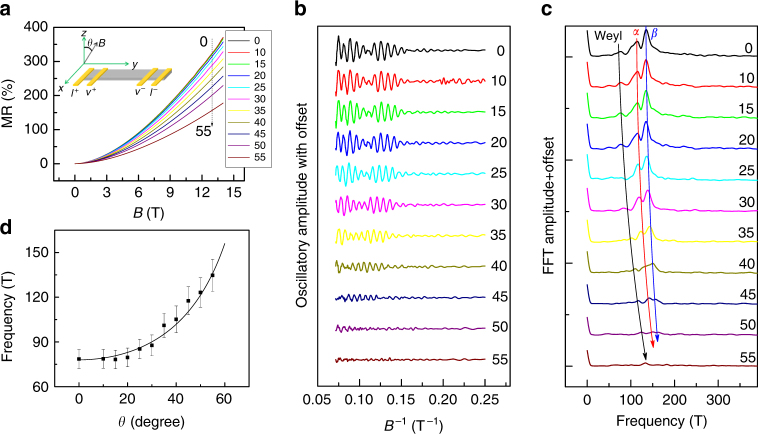
Angle-dependent quantum oscillations. **a** The MR data from *b*-axis ribbon obtained at different angles and *T = *2 K. **b** The d^2^ *R*/d*B* ^2^data obtained from **a** clearly shows quantum oscillations. **c** The evolution of quantum frequency in FFT spectra obtained from **b** as the angle increases. The arrow indicates the evolution of the peaks. **d** Angle dependence of extracted Weyl orbit frequency. It shows a $\cos ^{ - 1}\theta$ dependence (solid line), indicating a 2D surface state. The error bar of 20%, including standard instrument error and FFT frequency spacing estimate, was added into the angular dependence of frequency

As shown in Fig. 1b, the Weyl orbit can only be detected when a non-zero, perpendicular component of magnetic field is applied to the *ab* plane; therefore, some two-dimensional (2D) characteristics of the Weyl orbit should be observed. Fig. 2a shows that MR decreases significantly as the angle increases from *θ = *0° (**B**//*z*-axis) to *θ = *90° (**B**//*ab* plane). The SdH oscillations at different angles are clearly observed in the plots of d^2^ *R*/d*B* ^2^ vs. *B* ^−1^ (Fig. 2b). It is evident that the SdH oscillations evolve with an increasing *θ*. The frequencies of *α* and *β* increase with *θ*, as expected given the ellipsoid-like electron pockets in the bulk WTe_2_ (Supplementary Fig. 5). The most interesting feature in Fig. 2c is that the oscillation frequency of the Weyl orbit shifts faster than that of the two electron pockets, indicating that the Weyl orbit has different origin from that of the two electron pockets. The extracted extra frequency *F*_S_(*θ*) in Fig. 2d can be described by a $\cos ^{ - 1}\theta$ dependence, indicating that the Weyl orbit is 2D in nature. However, to observe the frequency of a Weyl orbit at higher angles (*φ* > 60°), the minimum magnetic field must be stronger than 17 T (~8.6 T/ cos 60), which exceeds the maximum magnetic field of 14 T in our experiment.

### Anisotropic chiral anomaly

**Fig. 3:**
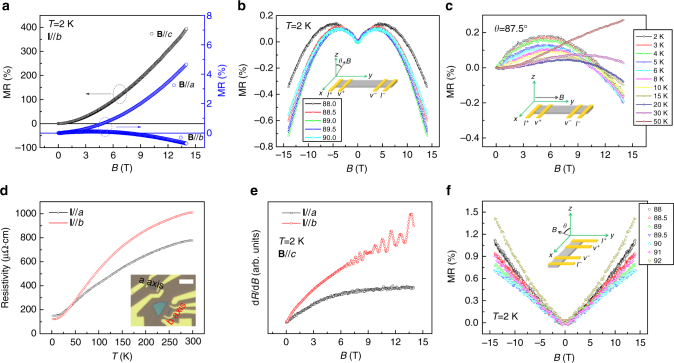
Chiral-anomaly induced negative longitudinal MR. **a** MR data obtained along different field directions at 2 K show strong anisotropy in the transport properties. In all of the measurements, **I**//*b* in the 19.4 nm thick *b*-axis ribbon. **b** The field-dependent, negative and longitudinal MR induced by a chiral anomaly in *b*-axis ribbon. We note that the occurrence of the maximum negative MR at 89° rather that at 90° could be due to a misalignment when we glued the sample to the sample holder. **c** The longitudinal MR measured with *θ = *87.5° at different temperatures. **d** The temperature-dependent resistivity of 19.4 nm thick *a*-axis and *b*-axis ribbon. The scale bar in the optical image is 5 μm. **e** Comparison of quantum oscillation in both ribbons with the magnetic field normal to the ribbon. **f** The absence of chiral-anomaly-induced negative MR in *a*-axis ribbon

Another crucial piece of evidence confirming the existence of type-II Weyl semimetals is the anisotropic negative longitudinal MR induced by a chiral anomaly. The negative longitudinal MR appears only when a magnetic field (*B*) are parallel to the tilt direction of Weyl cones, but chiral Landau levels, i.e., negative longitudinal MR, are absent when a magnetic field is normal to the tilt direction of Weyl cones^6,23–26,32^. The MR curves measured at 2 K with **B**//*a* and **B**//*b* are shown in Fig. 3a. The MR data for **B**//*c* (shown in Fig. 1d) is replotted here for comparison. Although the magnetic fields are perpendicular to the current in both cases, **B**//*a* and **B**//*c*, the positive MR ratio when **B**//*a* is almost two orders of magnitude smaller than that when **B**//*c*, which is consistent with a previous observation in bulk WTe_2_^33^. This strongly anisotropic MR behavior is ascribed to the strong anisotropy in the carrier mobility^28,33^. As expected, we observe a negative, longitudinal MR under high magnetic fields (*B *> 4 T) in the case of **B**//**I**//*b*, which strongly supports the prediction of an Adler–Bell–Jackiw anomaly in the presence of a strong magnetic field, and is in good agreement with recent works^22,23,34–36^. We carefully examined the origin of the negative MR and finally ruled out other possible mechanisms of negative MR, such as current jetting and Knudsen effect^37–40^ (see details in Supplementary Note 2 and Supplementary Figs. 9 and 10). To further confirm that the negative MR for **B**//**I**//*b* was indeed induced by a chiral anomaly, we measured the longitudinal MR as we slightly varied the applied field direction. As shown in Fig. 3b, the absolute value of the negative MR decreases quickly as the magnetic field deviates slightly from the *E*-field direction, a unique feature of a negative MR induced by a chiral anomaly^4^. The temperature-dependent MR measured at *θ = *87.5° (Fig. 3c) indicates clearly that the chiral anomaly can survive up to 30 K. The behavior of the magnetic field-dependent MR can be well described by the semi-classical model, in which both the chiral anomaly and the weak antilocalization (Supplementary Fig. 11) are considered^4^. To summarize, we have observed two unique properties of a type-II Weyl semimetal in the same piece of *b*-axis WTe_2_ ribbon: a quantum oscillation induced by a Fermi arc and a negative MR induced by a chiral anomaly. Therefore, we conclude that WTe_2_ is, indeed, a type-II Weyl semimetal.

One may argue that the observed quantum oscillation comes from the trivial surface states instead of a Weyl orbit (Fermi arcs). If the above observations really originated from the trivial surface states, we would be able to observe a similar quantum oscillation and negative MR induced by a chiral anomaly in an *a*-axis ribbon. To address this argument, we measured the magneto-transport properties on an *a*-axis ribbon fabricated from the same WTe_2_ slab used for the *b*-axis ribbon (Supplementary Fig. 4a). The temperature-dependent resistivity curves of the WTe_2_ nanoribbons are shown in Fig. 3d. A higher residual resistivity along the *a*-axis than along the *b*-axis indicates that the average carrier mobility is smaller along the *a*-axis than along the *b*-axis (*ρ* ^−1^ = (*n* + *p*)*eμ*), which is consistent with the transport anisotropy observed previously in bulk WTe_2_^28,33^. Although we observed a large, positive MR in the *a*-axis ribbon at low temperatures, similar to the *b*-axis sample (Supplementary Fig. 12), we were not able to observe any trace of quantum oscillation, even after removing the linear background (Fig. 3e). The complete disappearance of all quantum oscillations, including those arising from the intrinsic electron pockets of bulk sample, may be attributed to the strong mobility anisotropy *μ*_*a*_<*μ*_*b*_, which is supported by the observation in Fig. 3d ^28^. The disappearance of the quantum oscillation caused by a Weyl orbit in the *a*-axis ribbon can be ascribed to the fact that no Weyl orbit can be formed without the presence of Fermi arcs along the *X* direction in momentum space (Fig. 1a, b).

To further demonstrate that the Weyl points and Fermi arcs are found along the *Y* direction in WTe_2_, we measured the longitudinal MR of the *a*-axis ribbon at 2 K. As expected, no chiral-anomaly-induced negative MR was observed because the chiral Landau levels are absent^24–26^ when the magnetic field (**B**//*a*) is normal to the tilting direction of Weyl cone (*B*⊥*b* Fig. 3f). A similarly anisotropic behavior of the chiral anomaly was observed previously in WTe_2_ slab samples^34^ and was attributed to the fact that the kinetic energy is smaller than the potential energy, along the *a*-axis (*X* direction in momentum space)^6,22,34^. Therefore, the absence of both a Weyl orbit quantum oscillation and a chiral anomaly in *a*-axis ribbon further verifies the prediction that non-trivial topological Fermi arcs are only found along the *Y* direction in the (001) plane of WTe_2_.

### Thickness dependence of Weyl orbit

**Fig. 4:**
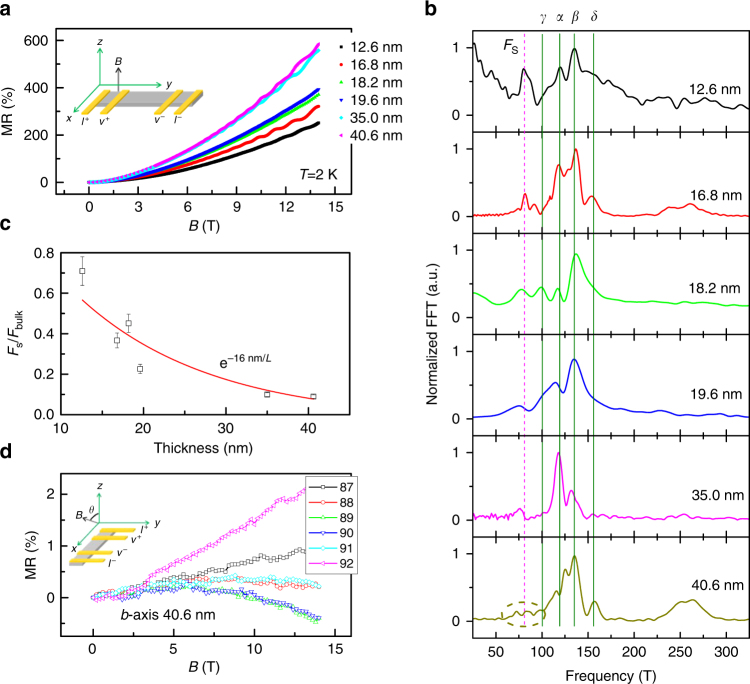
Thickness-dependent Weyl orbit oscillations. **a** The MR of *b*-axis ribbon with different thicknesses. **b** The FFT spectra of *b*-axis ribbons with different thicknesses, in which the spectra are normalized with respect to the strongest frequency of electron pocket *β*. The Weyl orbit frequency almost vanishes in 40.6 nm thick ribbon. **c** Thickness dependence of the relative amplitude of the Weyl orbit oscillation and the bulk oscillation with respect to peak *β*. The thickness dependence of relative amplitude can be fitted by $e^{ - L_0/L}$, with *L*_0_ = 16.0 nm. The fitted value of *L*_0_ is in the same magnitude of the mean free path (*c*-axis) ~48.0 nm. The 20% error bar was added given to the FFT frequency spacing estimate. **d** The chiral-anomaly-induced negative MR in 40.6 nm thick *b*-axis ribbon

As theoretically predicted^19^ and experimentally demonstrated^12^ previously, a key characteristic of the quantum oscillation from a Weyl orbit is that its amplitude decays very fast with increasing the ribbon thickness. Plotted in Fig. 4a are the MR data measured at 2 K from the *b*-axis ribbons with thickness varying from 12.6 to 40.6 nm. The MR increases greatly as the thickness of the sample increases. This enhanced MR in thicker ribbons can be ascribed to the increased mobility^27^ and the decreased Fermi level. Both factors lead to a larger *n*–*p* compensated MR^18,27^. To clearly demonstrate how the quantum oscillation of the Weyl orbit depends on the thickness of the ribbon, we normalized the FFT spectra with respect to the strongest bulk Fermi surface frequency (Fig. 4b). It is evident in Fig. 4b that the Weyl orbit frequency (~78 T) is very strong in the 12.6 nm sample, and it decays quickly as the thickness of the ribbon increases; nearly vanishing when the thickness is increased to 40.6 nm (dashed circle in Fig. 4b). This fast decay can be seen more clearly in Fig. 4c, in which the ratio of $F_{\mathrm{S}}/F_{\max }$ is plotted vs. thickness. Although the extra-quantum oscillation could hardly be observed in the thickest nanoribbon (40.6 nm), the negative MR induced by a chiral anomaly in **B**//**I**//*b* was large enough (about 0.5% at 14 T) to be detected (Fig. 4d). Even though it was smaller in the 40.6 nm thick nanoribbon than that in the 19.4 nm thick ribbon, the negative MR strongly indicates that the Weyl points along **Y** direction still play an important role in the chiral anomaly transport. To further support our conclusion that type-II Weyl points and a Fermi arc exist in WTe_2_, we analyzed the vanishing Weyl orbit quantum oscillation in more detail in the 40.6 nm thick sample. Given the Fermi velocity $\bar v_{\mathrm{F}} = 3.09 \times 10^5{\mathrm{ms}}^{{\mathrm{ - 1}}}$, the time required for an electron to travel between the top and bottom surfaces along the *c*-axis can be approximated as *t* ≈ *L*/*v*_F_ = 40.6 nm/3.09 × 10^5^ms^−1^ ≈ 1.3 × 10^−13^ s, which is quite close to the coherent quantum scattering time of both electron pockets, $\bar \tau = \left( {1.6 \pm 0.1} \right) \times 10^{ - 13}{\mathrm{s}}$ (Supplementary Fig. 8). Not surprisingly, the Fermi-arc-induced quantum oscillation at ~78 T vanishes (Fig. 4b) in the 40.6 nm thick *b*-axis ribbon. The fast decay of the Weyl orbit agrees well with both the theoretical predictions and the experimental results obtained from slabs of Cd_3_As_2_^12,19^. The thickness-dependent decay of amplitude of Weyl orbit could be reasonably well-fitted by exponential behavior ($e^{ - L_0/L}$) with *L*_0_ ≈ 16.0 nm, which is in the same magnitude of the mean free path *l* ≈48.0 nm(*c*-axis, $v_{\mathrm{F}}\bar t \approx 48.0{\mathrm{nm}}$).

## Discussion

Therefore, to observe the topologically protected Fermi-arc-induced quantum oscillation, the sample thickness must be large enough to ensure both metallic behavior (>10 nm) and SdH oscillation, and the time spent crossing the sample ribbon must not exceed the coherent quantum scattering time (Supplementary Figs. 13 and 14 and Supplementary Note 1). Although we can observe the decay of oscillation amplitude of Weyl orbit quantum oscillations on thickness, it is still difficult to observe the effect of thickness on Weyl orbit quantum oscillation phase^12^ (see Supplementary Note 3 and Supplementary Fig. 15). Finally, we have also examined if this additional quantum oscillation is a consequence of Klein tunneling, since it was predicted theoretically that electrons may tunnel through a single type-II Weyl point with the mechanism of Klein tunneling^41^. Unfortunately, we were unable to exclude or demonstrate the existence of this quantum oscillation (Supplementary Note 4 and Supplementary Fig. 16).

To conclude, although WTe_2_ was suggested to be a type-II topological Weyl semimetal, a full experimental confirmation of this theory has been lacking, owing to the limited resolution of ARPES. We have presented strong experimental evidence to confirm that WTe_2_ is indeed a type-II topological Weyl semimetal by observing two unique properties: an extra-quantum oscillation arising from a Weyl orbit and an anisotropic negative MR resulted from a chiral anomaly. Our work and the work on the Dirac semimetal Cd_3_As_2_, together with the theoretical prediction, have demonstrated that the magneto-transport measurements, including both the direct evidence, the negative longitudinal magnetoresistance induced by anisotropic chiral anomaly and the indirect evidence, Weyl orbit, are reliable and convenient to explore the existence of type-II Weyl semimetals.

## Methods

### Device fabrication

A schematic of the device fabrication process is shown in Supplementary Fig. 1. First, the multilayered WTe_2_ slabs were exfoliated from the CVD-grown single crystal, which was bought from Hqgraphene Company. The in-plane crystal orientation (*a-*axis and *b-*axis) was determined using the angle-dependent polarized microscopic Raman spectrum (Hariba LABRAM HR spectrometer), as shown in Supplementary Fig. 1b. An excitation laser with a wavelength of 473 nm was used. To avoid oxidation caused by the laser, a filter was inserted to make sure that the laser power was below 300 μW during the Raman measurement. To minimize oxidation from the surrounding air, the newly exfoliated WTe_2_ slabs on a SiO_2_(285 nm)/Si substrate were placed in a vacuum chamber immediately after the Raman measurement.

To measure the transport properties with current flowing along the *a* and *b* axes, WTe_2_ nanoribbons with widths of 0.6-1 μm and lengths of 10–15 μm were shaped using the standard E-beam lithography (EBL) process, which was followed by a reactive etching with Cl_2_ gas to remove the excess WTe_2_ slabs, as shown in Supplementary Fig. 1c. Finally, small electrodes Ti(10 nm)/Au(70 nm) were shaped using the EBL process again, which was followed by E-beam evaporation. Supplementary Fig. 1 includes a schematic of the nanofabrication method. Another slab (~700 nm, Supplementary Fig. 11) was fabricated using a focused ion beam attached to the scanning electron microscope (SEM, Helios 650, FEI).

### Transport measurement

The thickness of the WTe_2_ slabs and the ribbon-like slab were determined using an atomic force microscope (AFM). The morphology of the devices was characterized by scanning electron microscopy (SEM Nova Nano 630, FEI). The magneto-transport of the ribbon-like devices with different crystal configurations was measured in the physical properties measurement system (Dynacool) by quantum design, with a temperature range of 2–300 K and a magnetic field up to 14 T, during which a sample rotator helped to change the direction of the magnetic field with respect to the current. The resistance was measured using the ac lock-in method with the standard four-terminals. An AC current of 1 μA was applied to avoid overheating and damaging the nanoribbons. The magnetoresistance ratio of our samples was defined as MR = (*R*_*B*_ − *R*_0_)/*R*_0_. To analyze the quantum oscillation, we used d*R*/d*B* or d^2^ *R*/d*B* ^2^ to remove the large linear magnetoresistance background, which was caused by the nearly compensated *n*–*p* compensation in semimetallic WTe_2_^42^.

### Data availability

The data that support the findings of this study are available from the corresponding author upon request.

## Electronic supplementary material

Supplementary Information

Peer Review File
