# Supplementary material for: Evidence for topological type-II Weyl semimetal WTe2
Source: Nat Commun. 2017 Dec 15;8:2150. doi: 10.1038/s41467-017-02237-1 (PMC5732285; doi:10.1038/s41467-017-02237-1)
Supplement: Supplementary file 1 — Supplementary Information [file 41467_2017_2237_MOESM1_ESM.pdf]

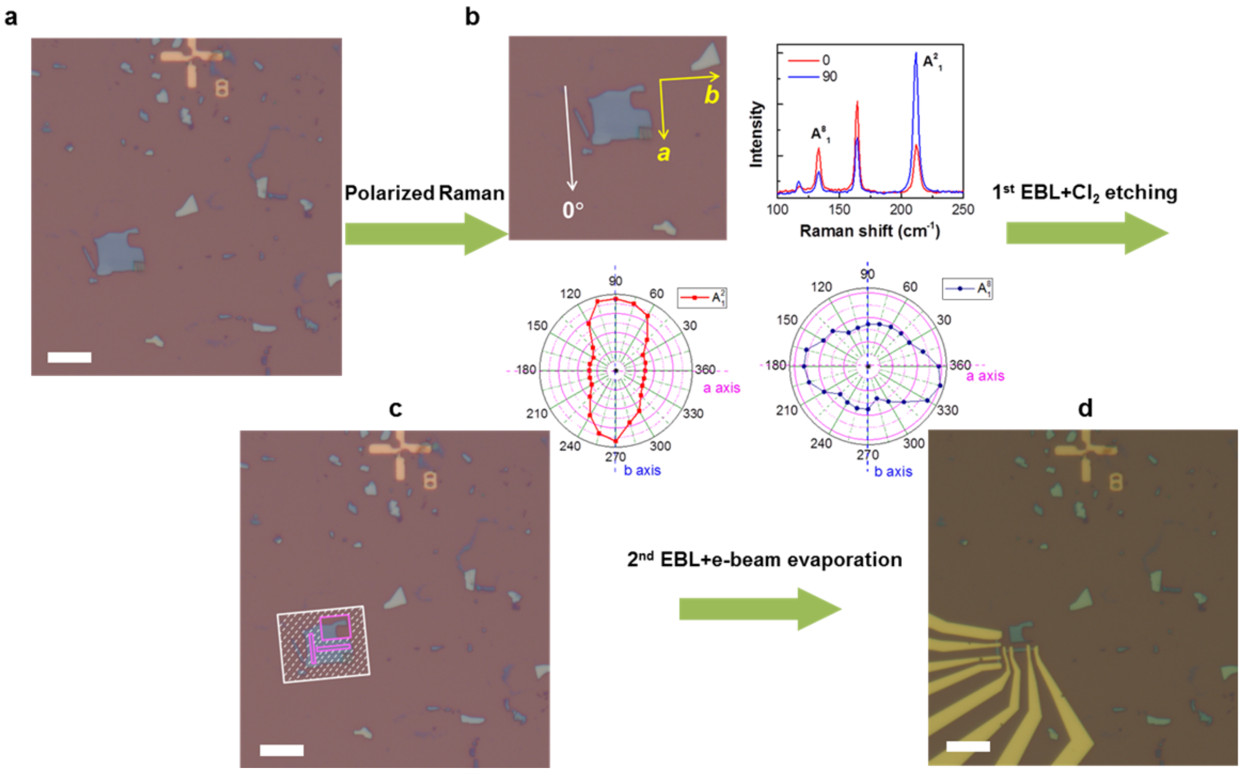

Supplementary Figure 1| Schematic of WTe_2 nanoribbon fabrication with different crystal directions. a, Exfoliated WTe_2 slabs on SiO_2(285 nm)/Si substrate. **b**, In-plane crystal direction as determined by polarized Raman. The angle is indicated by the white arrow in optical image. The intensity of the Raman spectra collected at θ = 0° and θ = 90° are significantly different, especially for the vibration modes A_1^8 (~130 cm^{-1}) and A_1^2 (~212 cm^{-1})[1-3]. During the polarized Raman measurement, the incident polarization vector $\mathbf{e}_i$ was parallel to the scattered polarization vector $\mathbf{e}_s$. While the incident polarization vector $\mathbf{e}_i$ and scattered polarization vector $\mathbf{e}_s$ were parallel to the in-plane *a*-axis, the Raman intensity of A_1^8 and A_1^2 was maximized and minimized, respectively[1,4]. Therefore, we easily determined the in-plane crystal direction through the extracted angle-dependent Raman intensity of A_1^8 and A_1^2. **c**, After the first EBL writing, the area shaded with white dashes was exposed by the electron beam, leaving the area inside the purple rectangle protected by photoresist. Then, the excess WTe_2 slab under dashed lines was

reactively etched with Cl_2 in 15 seconds. **d**, The final device with Ti/Au electrodes was fabricated by a second EBL writing, which was followed by E-beam evaporation and standard lift-off with acetone. The white scale bar in all optical images is 10 μm.

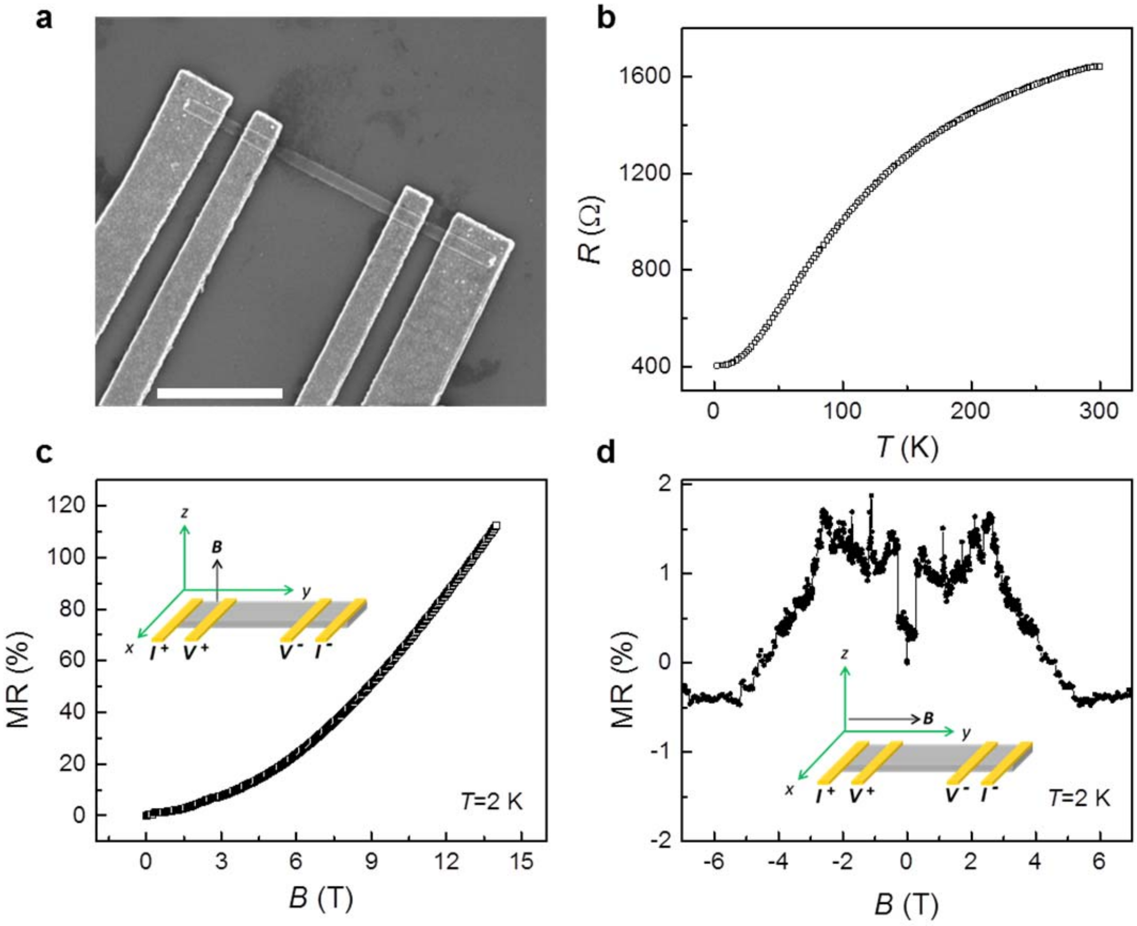

Supplementary Figure 2| Universal conductance fluctuation in narrow nanoribbon sample. **a**, Scanning electron microscope (SEM) image of *b*-axis ribbon with a width of ~110 nm. The white scar bar is 1 μm. **b**, Temperature dependence of resistance shows a metallic behavior. **c**, Moderate MR ratio of 110% at 14 T and 2 K for the magnetic field normal to the nanoribbon. **d**, The noisy longitudinal MR in ~110 nm wide ribbon.

This noisy MR at 2 K is nearly symmetric for the positive and negative magnetic field regions, which is a strong sign of universal conductance fluctuation[5]. To obtain a much smoother and more reliable quantum oscillation without the extrinsic effect, we fabricated the nanoribbon with a width larger than 600 nm in all of the experiments.

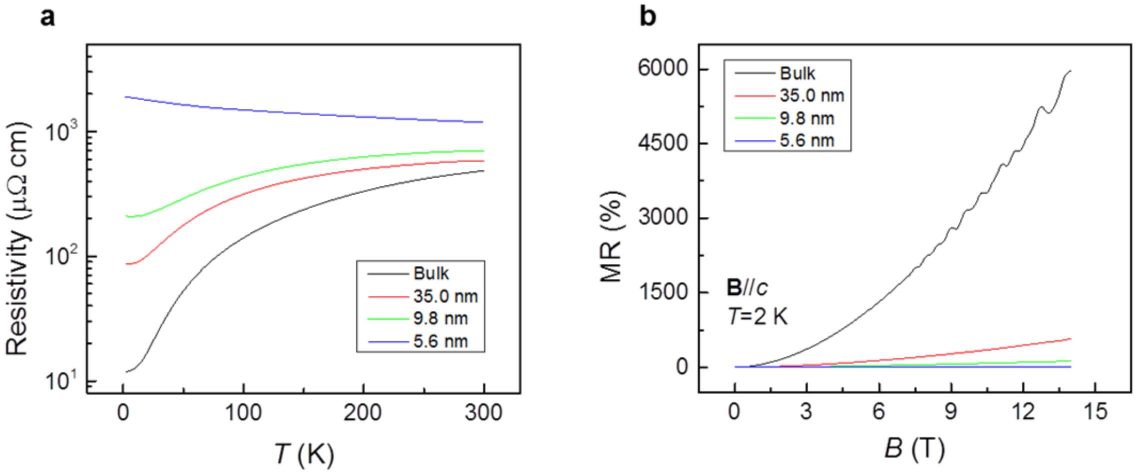

Supplementary Figure 3 | Metal-insulator transition in WTe_2 with few layers. a, Temperature-dependent resistivity of WTe_2 bulk and slabs with different thicknesses. **b**, Suppression of *n*-*p*-compensated MR in atomically thin WTe_2 slabs, compared to the rather large MR ratio in bulk WTe_2.

As the thickness decreases, the metallic behavior of WTe_2 slab becomes semiconductive, indicating a band-gap opening in ultrathin samples.[6] To obtain the quantum oscillation information of samples with different in-plane crystal directions, we fabricated the ribbon with a thickness thicker than 10 nm in all of the experiments.

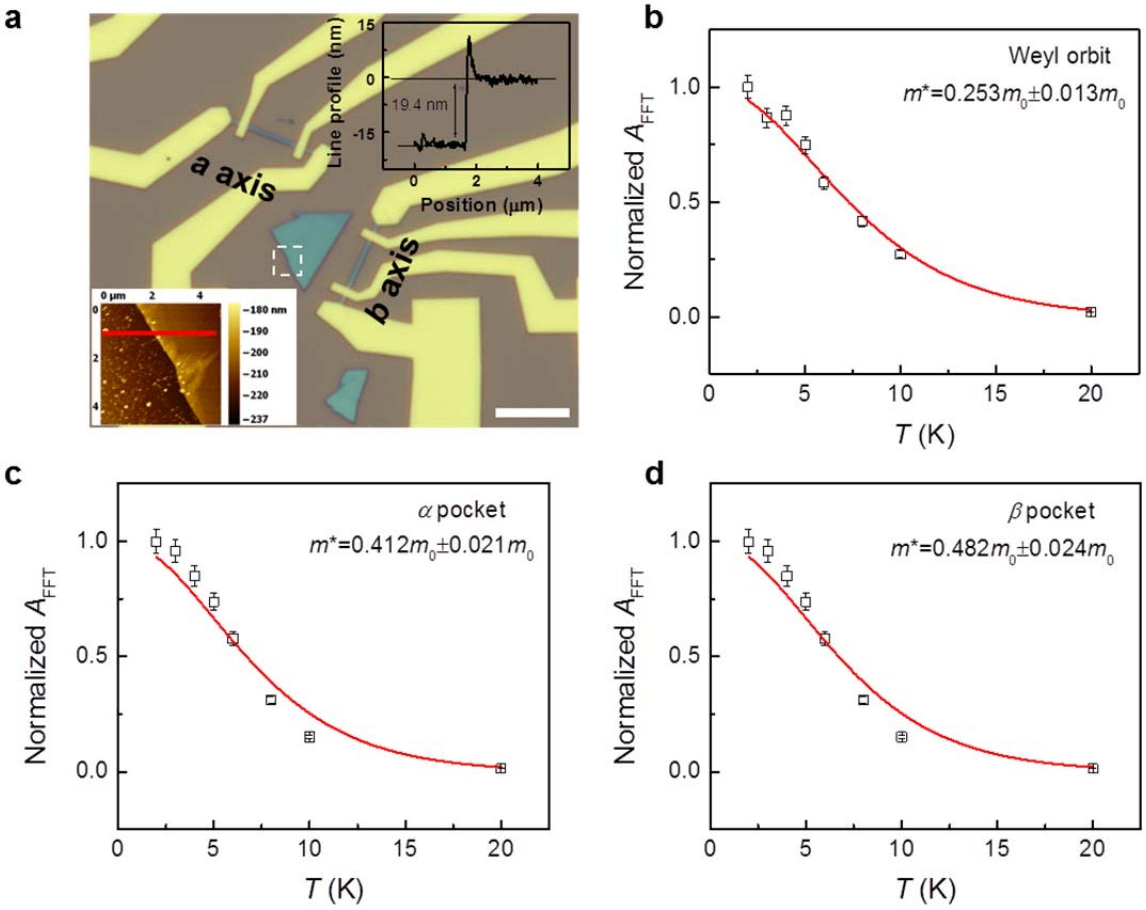

Supplementary Figure 4| Effective mass analysis of two electron pockets of WTe_2 and Weyl orbit oscillation. a, Optical image of nanoribbon devices; the inset contains an AFM image of the area indicated by the white dashed square and a profile line scan, which determines the thickness of nanoribbon to be 19.4 nm. The scale bar is 10 μm. A_{FFT} is the amplitude of FFT spectrum. The extracted temperature-dependent fast Fourier transformation (FFT) amplitude of the Weyl orbit oscillation and two electron pockets (α and β) from the bulk WTe_2 are shown in **b**, **c** and **d**, respectively. We add the instrument error 10% into the data of **b**-**d**.

The fast decay of the FFT amplitude with temperature follows the Lifshitz-Kosevich equation[7],

$$\frac{\Delta\rho}{\rho} \propto \frac{X}{\sinh X} e^{-X_{\mathrm{D}}} \cos 2\pi[\frac{B_{\mathrm{F}}}{B} + \frac{1}{2} + \beta], \tag{1}$$

with $X = 2\pi^2 k_{\mathrm{B}} T m^* / \hbar eB = 2\pi^2 k_{\mathrm{B}} T / \hbar\omega_{\mathrm{c}}$ (Landau level difference $\hbar\omega_{\mathrm{c}} = \hbar eB / m^*$) and $X_{\mathrm{D}} = 2\pi^2 k_{\mathrm{B}} T_{\mathrm{D}} m^* / \hbar eB = 2\pi^2 k_{\mathrm{B}} T_{\mathrm{D}} / \hbar\omega_{\mathrm{c}}$, where m^* is the cyclotron effective mass of the carriers, $T_{\mathrm{D}} = h / 4\pi^2 \tau k_{\mathrm{B}}$ is the Dingle temperature and τ is the quantum lifetime of the carriers due to scattering. We fit the normalized FFT amplitude by $X / \sinh X$ in Supplementary Eq. (1), leading to the effective masses 0.253 m_0 (Weyl orbit), 0.412 m_0 (α) and 0.482 m_0 (β), as shown in **b**, **c**, and **d**. m_0 is the electron mass.

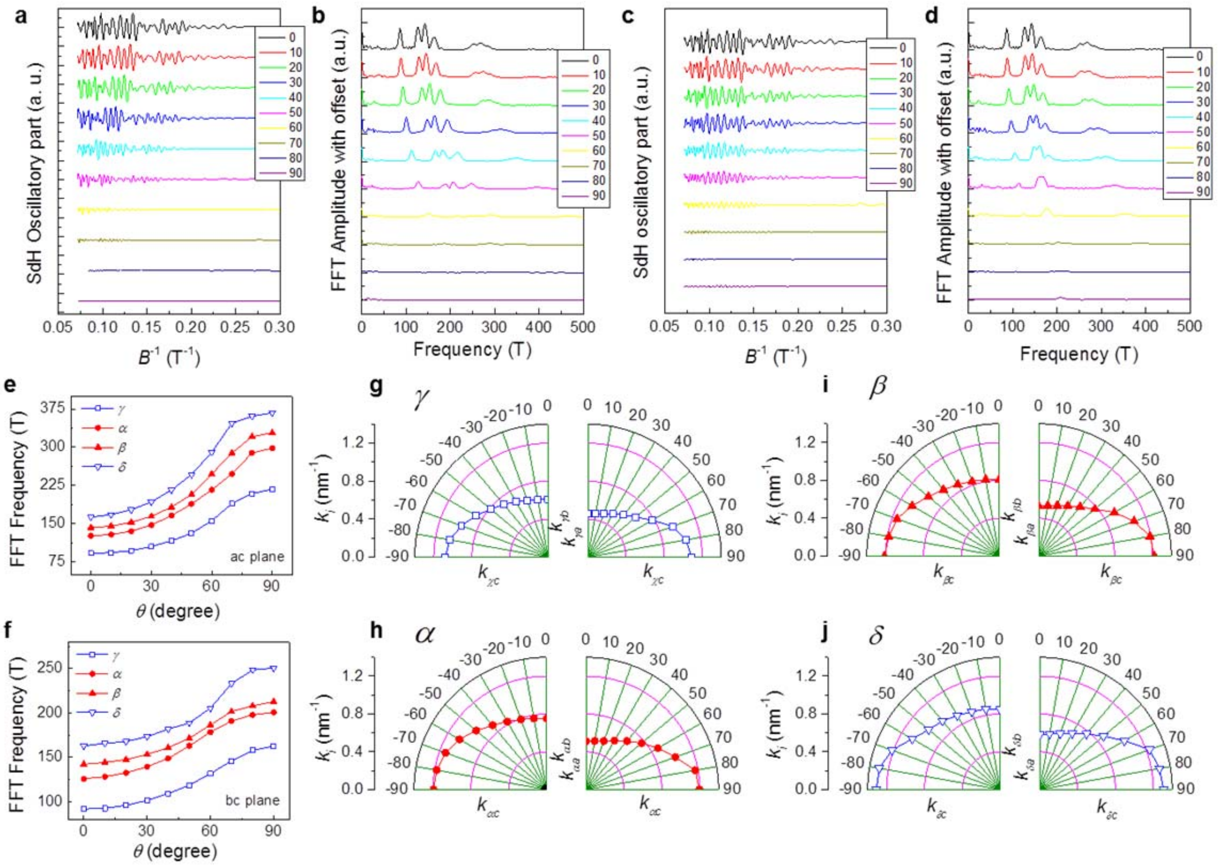

Supplementary Figure 5 Fermi surface mapping of bulk WTe_2. **a**, SdH oscillation with magnetic field rotation in *ac* plane; $\theta = 0°$ corresponds to the direction of the magnetic field parallel to *c*-axis. **b**, FFT spectra of SdH oscillations in *ac* plane. **c**, SdH oscillation with magnetic field rotated in *bc* plane; $\theta = 0°$ corresponds to the direction of the magnetic field parallel to *c*-axis. **d**, FFT spectra of SdH oscillations in *bc* plane. The extracted oscillation frequency of four pockets (two electrons, α and β; and two holes, γ and δ) is given in **e**, (*ac* plane) and **f**, **(***bc* plane**)**. To calculate the detailed Fermi surface information, we used the Onsager equation $F = (\hbar / 2\pi e) S_F$ [7], where S_F is the cross-sectional area in *k*-space. The four Fermi surfaces are triaxial ellipsoidal rather than spherical, as shown in **e** and **f**. The frequency is

$F_k = \frac{\hbar}{2\pi e}\pi k_i k_j = \hbar k_i k_j / 2e$ ($F_a = \frac{\hbar}{2e} k_b k_c$, $F_b = \frac{\hbar}{2e} k_a k_c$, $F_c = \frac{\hbar}{2e} k_a k_b$). Therefore, we calculated the Fermi surface dimensions of four pockets, as listed in Supplementary Table 1. The calculated cross-sectional image of each Fermi pocket is plotted in **g-j**. The contribution of each Fermi pocket to the carrier density can be calculated according to $n = \frac{V}{N} = 2 \times \frac{1}{(2\pi)^3} \times \frac{4\pi}{3} k_a k_b k_c$; see Supplementary Table 1. The data obtained from our sample agrees well with previous work[8]. This information about the Fermi surface comes from the magneto-transport data measured in the bulk WTe_2, which was prepared by focused ion beams (FIB) with a dimension of 10 μm × 2.5 μm × 0.7 μm ($a \times b \times c$). All the data are obtained at 2 K.

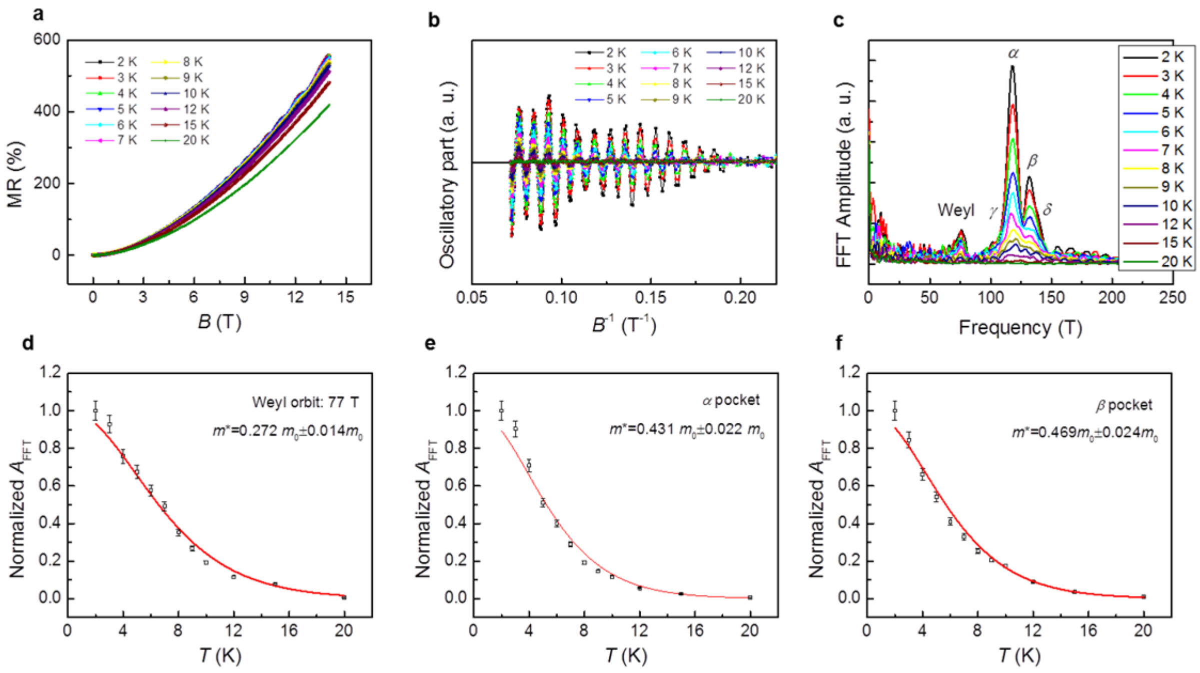

Supplementary Figure 6| Weyl orbit quantum oscillation and effective mass of WTe_2. Reproducible new Weyl orbit frequency and effective mass in *b*-axis nanoribbon with thickness *t*=35.0 nm. **a**, Field dependent MR at various temperatures, **b**, SdH oscillations in the plots of $d^2R/dB^2 \sim B^{-1}$, **c**, The FFT spectra at various temperatures. The temperature dependence of normalized FFT amplitude and fitting of effective mass. **d**, Weyl orbit, (**e**, α pocket, **f**, β pocket. A_{FFT} is the amplitude of FFT spectrum. We add the instrument error 10% into the data of **d**-**f**.

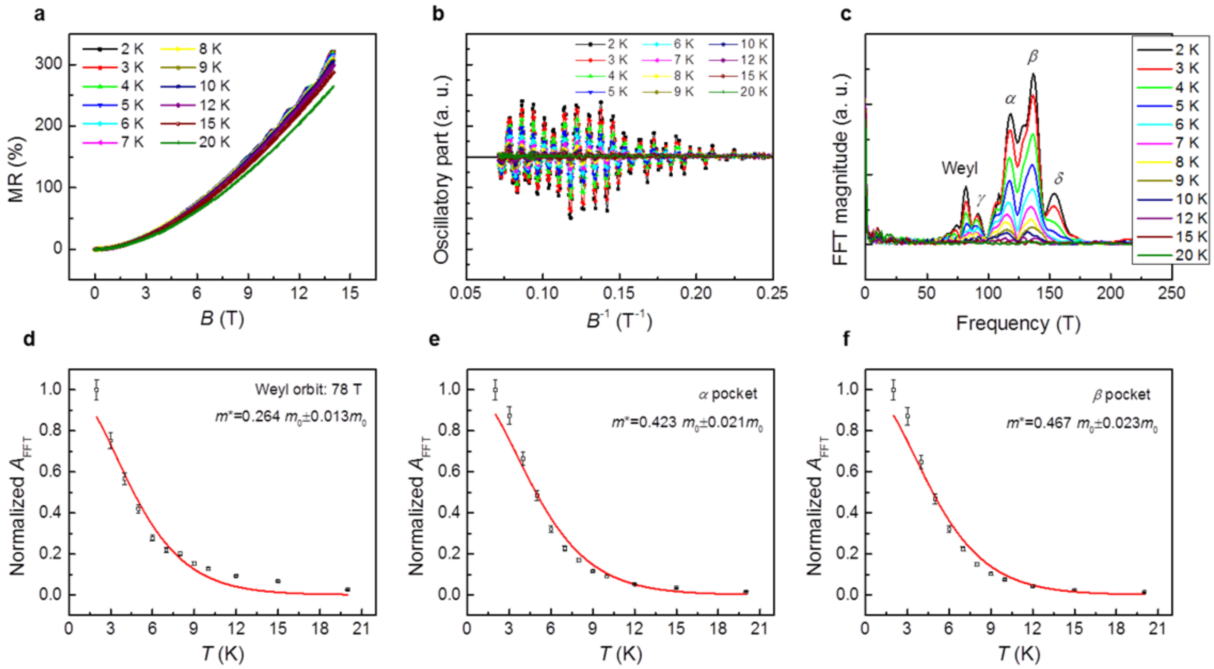

Supplementary Figure 7| Weyl orbit quantum oscillation and effective mass of WTe_2. Reproducible new Weyl orbit frequency and effective mass in *b*-axis nanoribbon with thickness t=16.8 nm. **a**, Field dependent MR at various temperatures, **b**, SdH oscillations in the plots of $d^2R/dB^2 \sim B^{-1}$, **c**, The FFT spectra at various temperatures. The temperature dependence of normalized FFT amplitude and fitting of effective mass. **d**, Weyl orbit, **e**, α pocket, **f**, β pocket. A_{FFT} is the amplitude of FFT spectrum. We add the instrument error 10% into the data of **d**-**f**.

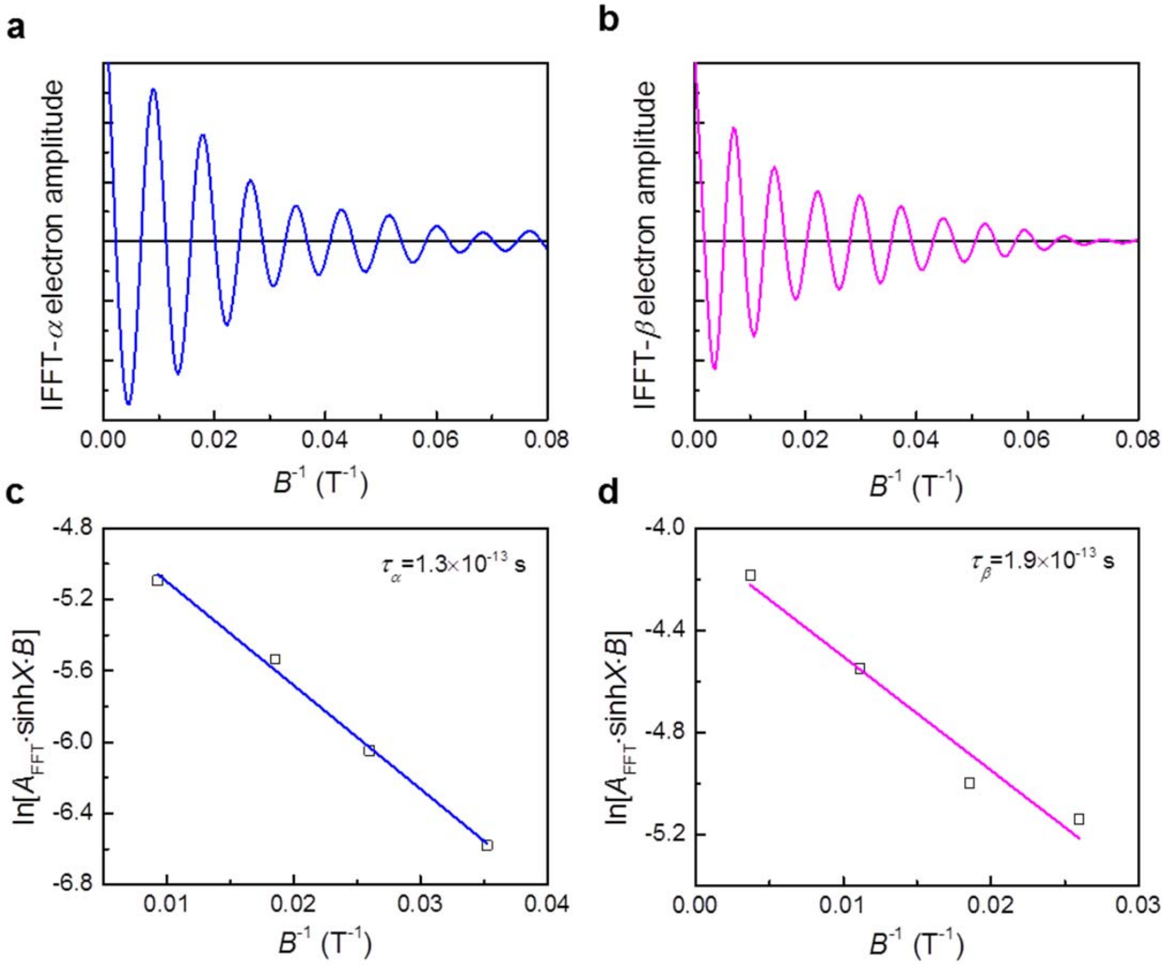

Supplementary Figure 8| Dingle plot of two electron pockets. a, Inverse FFT spectrum of single frequency of electron pocket α; the raw data is found in Fig. 1f. **b**, Inverse FFT spectrum of single frequency of pocket β. According to Supplementary Eq. (1), the linear fitting of $A_{FFT}B\sinh X$ to B^{-1} gives the quantum scattering time of electron pockets as **c**, α ($\tau_\alpha = 1.3\times10^{-13}\,\mathrm{s}$) and **d**, β ($\tau_\beta = 1.9\times10^{-13}\,\mathrm{s}$). A_{FFT} is the amplitude of FFT spectrum. Therefore, the average scattering time of both electron pockets is $\overline{\tau} = 1.6\times10^{-13}\,\mathrm{s}$.

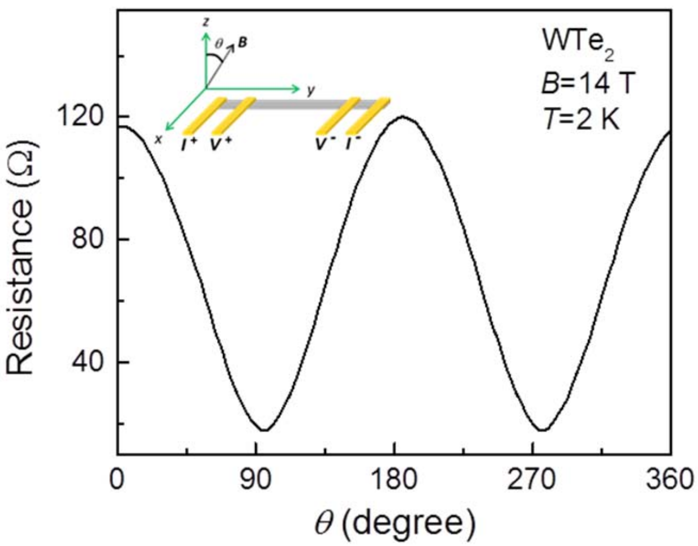

Supplementary Figure 9| Exclusion of current jetting effect in our devices. Typical angular dependence of longitudinal resistance of *b*-axis nanoribbon is measured under a magnetic field of 14 T and 2 K. The inset gives the measurement configuration.

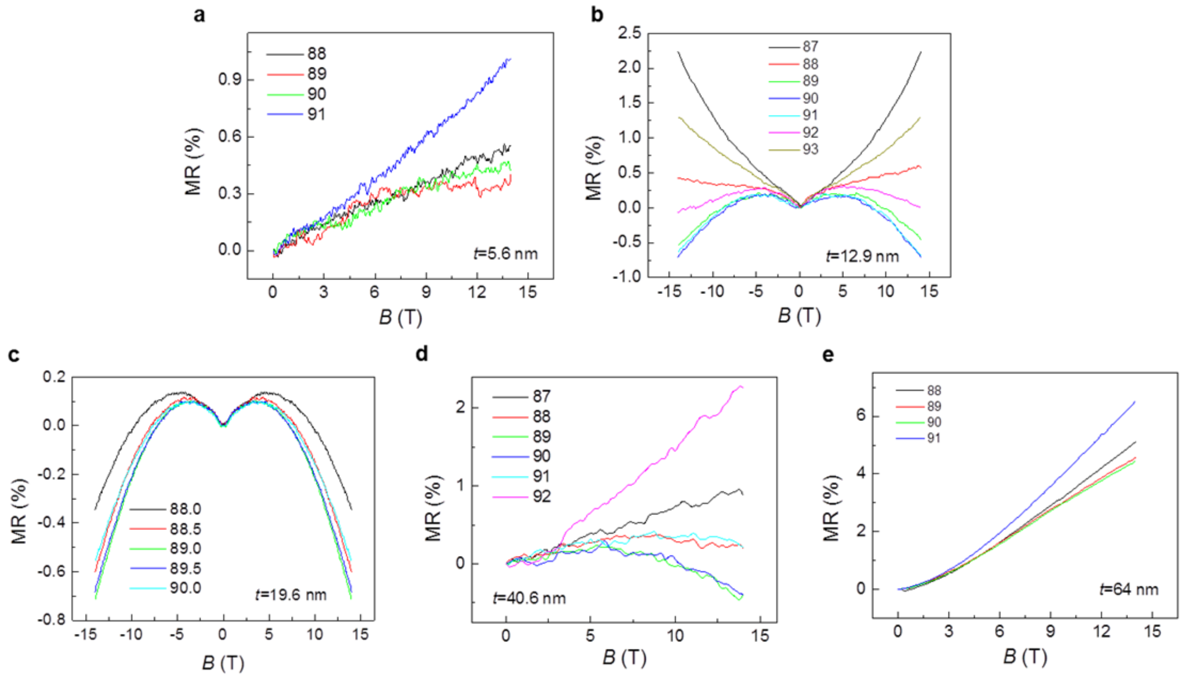

Supplementary Figure 10| Exclusion of Knudsen effect in our devices. Chiral anomaly induced negative magnetoresistance in the *b*-axis nanoribbons with various thicknesses, **a-e**. All the data were obtained at 2 K.

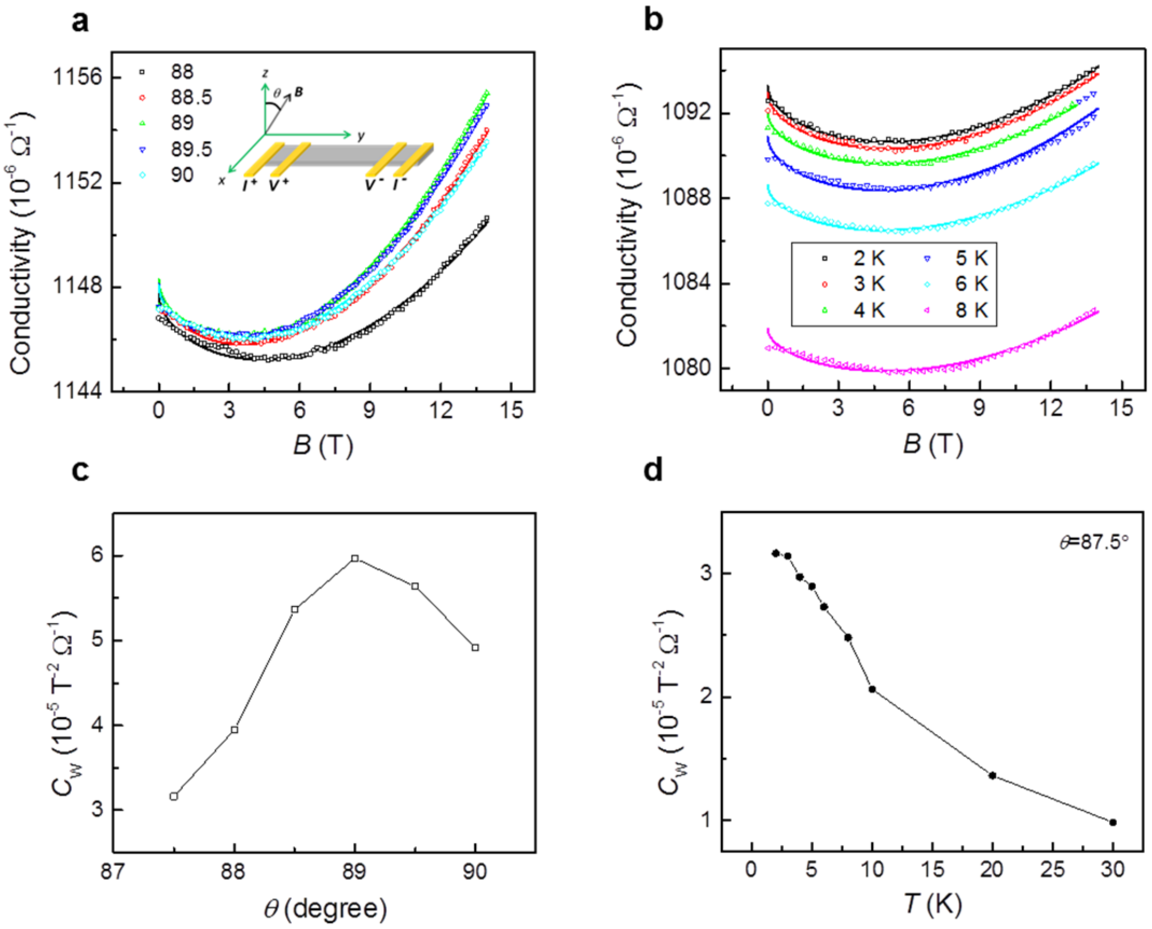

Supplementary Figure 11| Fitting longitudinal magnetoresistance by chiral anomaly. a, Magnetic field dependence of conductivity of *b*-axis ribbon at different angles (T = 2 K, t=19.4 nm). The chiral-anomaly-induced positive conductivity can be fitted by [9,10]

$$\sigma(B) = (1 + C_{\mathrm{W}} B^2) \cdot (\sigma_0 + a\sqrt{B}) + \sigma_{\mathrm{N}} \,. \qquad (2)$$

$C_{\mathrm{W}}B^2$ is the contribution of the chiral current with a positive value, $a\sqrt{B}$ is the conductivity contribution from weak antilocalization with a negative value and σ_{N} is the conventional nonlinear band contribution around the Fermi level. The solid lines indicate the fitting by Supplementary Eq. (2). **b**, Magnetic field dependence of conductivity at different temperatures (θ

= 87.5°). The solid lines also give the fitting by Supplementary Eq. (2). The fitted parameter C_W is listed in **c** and **d** as a function of angle and temperature, respectively. As shown in **c**, C_W reaches its maximum at the exact angle (**B**//**E**), and decays quickly as temperature increases.

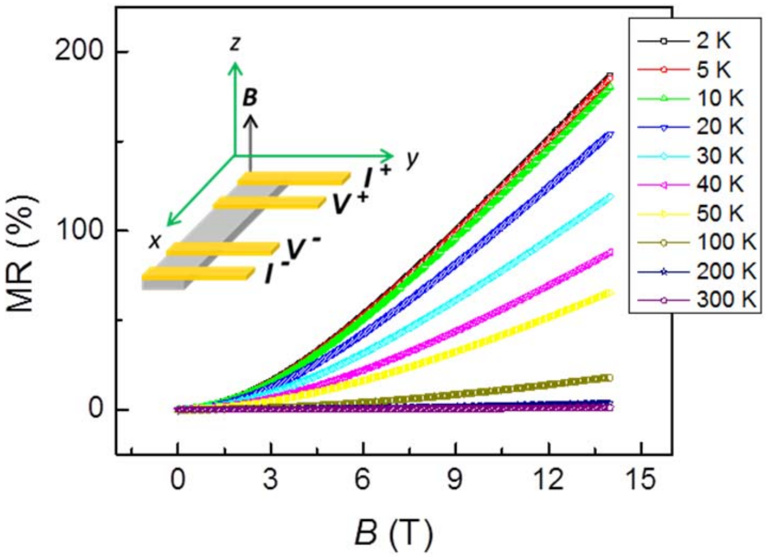

Supplementary Figure 12| Magnetoresistance of *a*-axis ribbon at different temperatures. The magnetoresistance decreases as the temperature increases in *a*-axis ribbon with a thickness of 19.4 nm. The inset gives the measurement configuration setup.

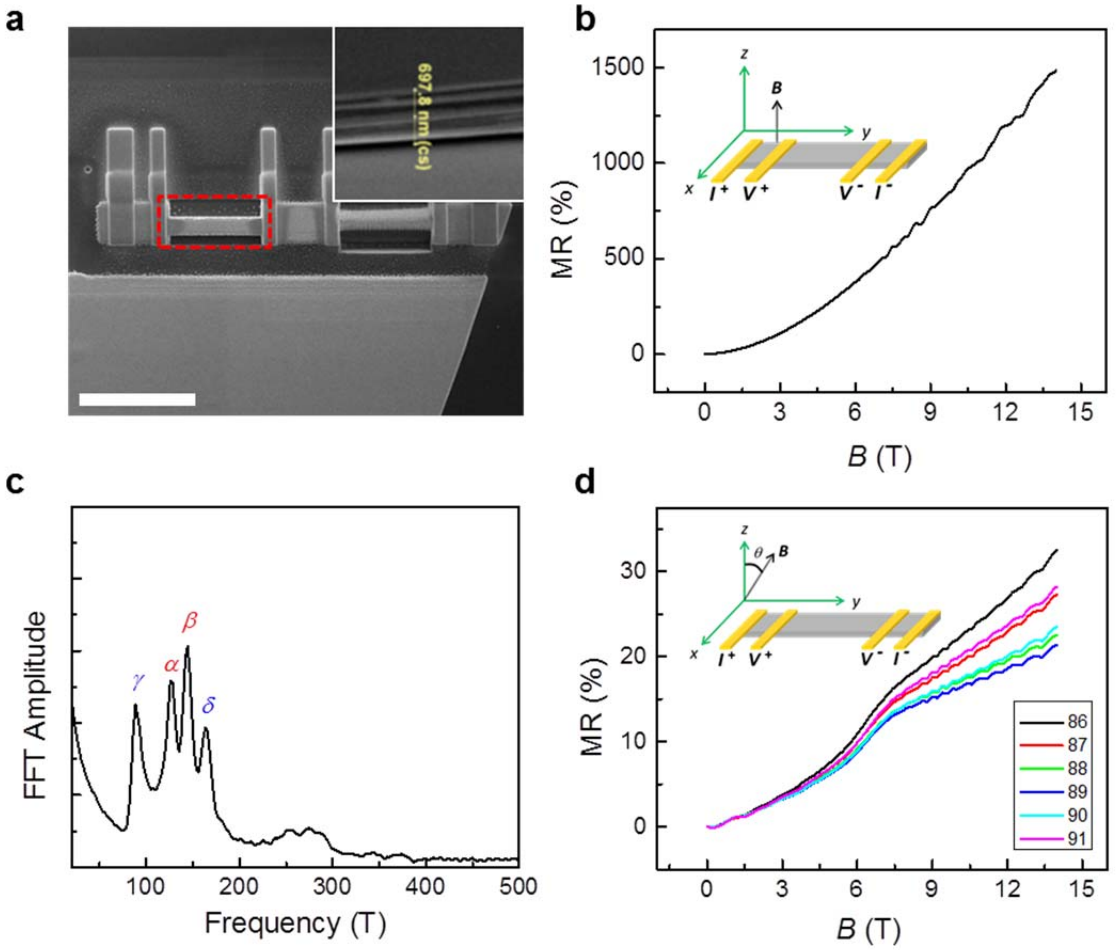

Supplementary Figure 13| Magneto-transport property of thick *b*-axis ribbon a, SEM image of *b*-axis ribbon, which was fabricated by FIB. The inset gives the tilt scan of this slab and its thickness of ~700 nm. The white scale bar is 10 μm. **b**, Magnetoresistance of this *b*-axis ribbon with **I**//*b*, **B**//*c*. **c**, FFT spectrum of the data in **b**. Only four Fermi pocket frequencies are observed in bulk WTe_2. The frequency near ~250 T comes from the superposition of individual frequency[8]. No Weyl orbit frequency is detected in this thick ribbon. **d**, Longitudinal MR of thick *b*-axis ribbon. No chiral-anomaly-induced negative MR is observed. All the data above are measured at 2 K.

In this much thicker *b*-axis sample (~700 nm thick), we observed only four frequencies, originating from two electron pockets and two hole pockets in the bulk WTe_2, without any trace of Fermi-arc-induced quantum oscillation around 80 T (**c**). While the longitudinal MR of thin nanoribbons is negative (Figs. 3b and 4d), the longitudinal MR of the 700 nm thick slab is positive, and significantly large (**d**). The disappearance of the negative longitudinal MR and the Fermi-arc-induced quantum frequency indicates that the Fermi level was far away from the location of the Weyl points in this thick sample. We fabricated the *a*-axis ribbon and the *b*-axis ribbon from the same slab using FIB. The oscillation frequency and longitudinal MR of the *a*-axis ribbon show no significant differences from those of the *b*-axis sample.

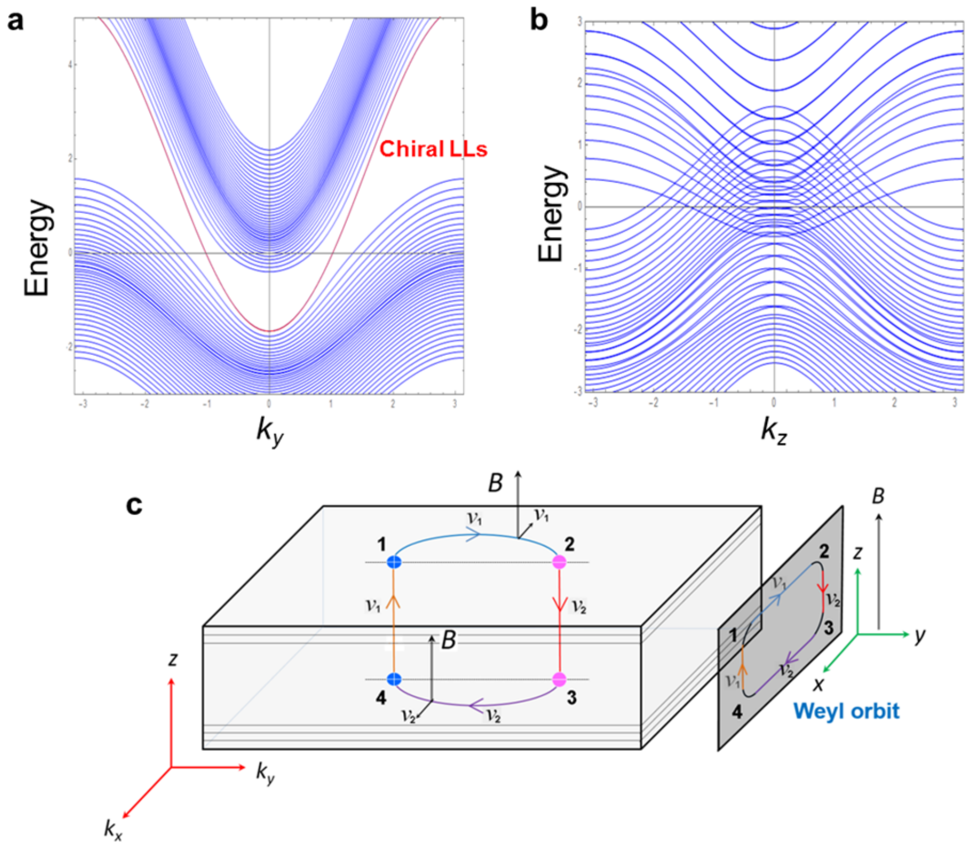

Supplementary Figure 14| Calculated Landau levels and Modified Weyl orbit in type-II Weyl semimetals. In the calculation, the Weyl cone tilts and separates along **Y** direction. **a**, A magnetic field is applied parallel to the tilting direction of Weyl cones (**Y** direction), i.e. **B**//*b*; **b**, A magnetic field is applied normal to the tilting direction of Weyl cone, i.e. **B**//*c*. The chiral Landau levels (LLs) are still visible in **a**. **c**, The modified Weyl orbit in mixed real and momentum space. Weyl orbit are consisted of four paths, two Fermi arcs (12 and 34), two bulk Landau level states with opposite Fermi velocity (23 and 14). The side view give the electron trajectory in real space, which is the same as the model proposed by A.C. Potter.[16]

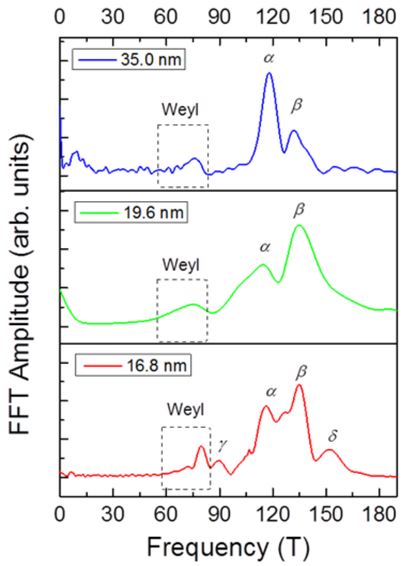

Supplementary Figure 15| Asymmetric Weyl orbit peak related to the non-adiabatic corrections. The Weyl orbit peak of the samples with different thicknesses shows asymmetric broadening towards low frequency due to the non-adiabatic correction, as the dashed rectangles shown.

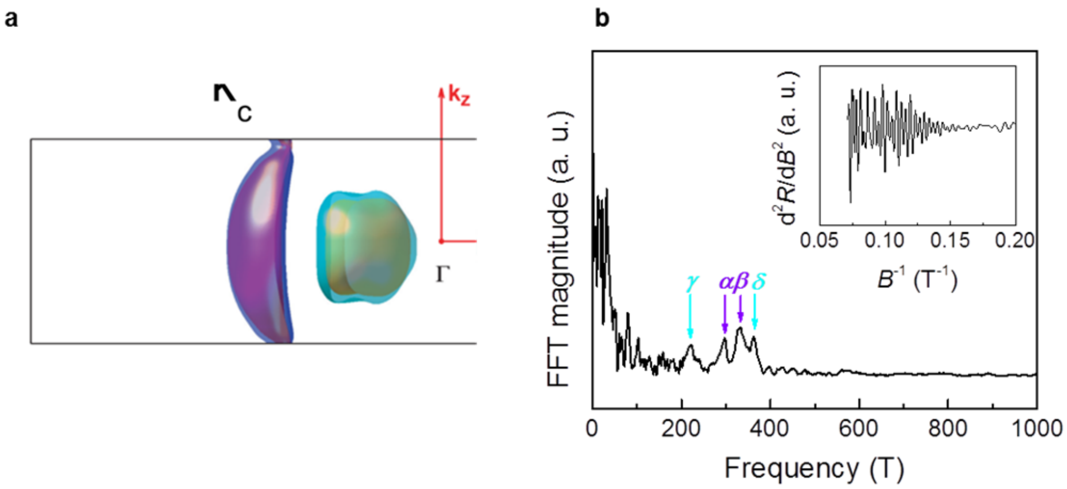

Supplementary Figure 16| Check for Klein tunneling in bulk WTe_2. a, The bulk Fermi surfaces of WTe_2 from previous work[8]. The light blue surface is hole pocket, and purple surface is electron pocket. **b**, The FFT spectrum of bulk WTe_2 with the magnetic field along *a*-axis of WTe_2. The inset gives the SdH oscillation at 2 K.

Supplementary Table 1| Detailed dimensions of four Fermi pockets in WTe_2.

	F_a (T)	F_b (T)	F_c (T)	k_a (nm^{-1})	k_b (nm^{-1})	k_c (nm^{-1})	n (10^{-19} cm^{-3})
γ (h)	217.6	162.6	92.0	0.46	0.61	1.08	1.02
α (e)	298.0	200.6	126.0	0.51	0.75	1.19	1.54
β (e)	338.2	212.8	142.2	0.53	0.81	1.22	1.77
δ (h)	367.5	250.5	162.9	0.58	0.85	1.31	2.18

Supplementary Note 1. Modified Weyl orbit model in type-II Weyl semimetals.

A number of theoretical and numerical works on the Landau levels in type-II Weyl semimetals[11-13] showed that the chiral Landau levels are still visible when the magnetic field is applied along the tilting direction of Weyl cone. Motivated by these work, we calculated the Landau levels in type-II Weyl semimetals for the exact same configurations as that in WTe_2: Weyl cone tilting along **Y** direction,[14,15] Weyl points separating along **Y** direction, the magnetic field applied along *b*- (**B**//*b*, Supplementary Figure 12a) and *c*-axis (**B**//*c*, Supplementary Figure 12b). Supplementary Figure 12shows the Landau levels with different magnetic field configuration. When the applied magnetic field is parallel to *b*-axis, i.e. **B**//*b*, we found that the chiral Landau levels are still well-defined (Supplementary Figure 12a), which is consistent with recent calculations.[11-13] This is why we can still observe the chiral anomaly induced negative longitudinal magnetoresistance in *b*-axis WTe_2 nanoribbons (Fig. 3b&3c). Whereas, when the applied magnetic field is normal to the tilting direction of Weyl cones the chiral Landau levels are missing (Supplementary Figure 12b), which agree well with the recent calculations as well.[11-13] In Dirac and type-I Weyl semimetals, the chiral Landau levels ($n = 0$) act as the only one-way "conveyor-belt", because the bulk Landau levels near the Fermi level in Dirac and type-I Weyl semimetals are gapped.[16] However, in type-II Weyl semimetals, when the magnetic field is normal to the tilting direction of Weyl cone, i.e. in WTe_2 **B**//*c*, the electron and hole pockets are not gapped and there are still several bulk Landau level states ($n \geq 1$) with opposite Fermi velocity near the Fermi level (Supplementary Figure 12b). Therefore, in a type-II Weyl semimetal, when the electrons arrive the Weyl points through topological Fermi arc states, they can traverse the nanoribbon via bulk Landau levels ($n \geq 1$) near the Weyl points. The bulk Landau levels ($n \geq 1$) with opposite Fermi velocities (up and down) can act as the paths to

transport the electrons between the top and bottom surfaces (Supplementary Figure 12c). Therefore, the Weyl orbit forms in type-II Weyl semimetals, in which two Fermi arcs and two bulk Landau levels ($n \geq 1$) with opposite Fermi velocities are involved.

After the slight modification on the Weyl orbit, we should notice that there are a lot of bulk Landau level states ($n \geq 1$) near the Fermi level. One will naturally ask a question. Will the scattering between the bulk Landau levels ($n \geq 1$) be too strong to destroy the Weyl orbit? To answer this question, we have carefully examined the time elapsed between the top surface and bottom surface. Fortunately, the Fermi velocity of the bulk electrons along *c* direction in our WTe_2 is as high as $\overline{v}_F = \hbar \overline{k}_c / \overline{m}^* \approx 3.09 \times 10^5 \, \mathrm{m\,s^{-1}}$ (Supplementary Figures 4 and 5), the mean free path for the bulk electron is around $l = v_F \overline{\tau} \approx 48 \, \mathrm{nm}$, if coherent scattering time of both electron pockets $\overline{\tau} \approx (1.6 \pm 0.1) \times 10^{-13} \, \mathrm{s}$ (Supplementary Figure 8) is considered. This mean free path is even longer than the thickness of the thickest sample (~35 nm) in which we could observe the Weyl orbit as shown in Fig. 4. Therefore, the electrons will finish the transport between two Fermi arcs before it was scattered off by other bulk Landau levels near Fermi level. Hence, when the magnetic field is applied normal to the tilting direction of Weyl cone (i.e. **B**//*c*), a Weyl orbit will be formed in a type-II Weyl semimetal only if the bulk Landau levels can substitute the missing chiral Landau levels.

Now, let's examine the time spent on each path and to find the Weyl orbit frequency. Supplementary Figure 12c shows the Weyl orbit in a type-II Weyl semimetal that is composed of two Fermi arcs (momentum trajectories) and two bulk Landau levels (real space trajectories). The velocities of the bulk Landau levels (positive and negative Fermi velocity) in type-II Weyl semimetals should be slightly different due to the different slops of the energy spectra (Supplementary Figure 12b). We define the velocity on path 41 and 12 is v_1, and the velocity on

path 23 and 34 is v_2. The time sliding along Fermi arc on top (bottom) surface is $t_{\text{arc top}} \approx k_0 / ev_1 B$ ($t_{\text{arc bottom}} \approx k_0 / ev_2 B$). The propagating time between top and bottom surface is $t_{\text{bulk 23}} \approx L / v_2$ ($t_{\text{bulk41}} \approx L / v_1$, L is the thickness of nanoribbon). Therefore, The total time completing a Weyl orbit is $t = (v_1 + v_2)(L + \frac{k_0}{eB}) / v_1 v_2$. Given the average velocity along c is $\bar{v}_{\text{F}} = 2v_1 v_2 / (v_1 + v_2)$ and the semi-classic quantum orbit requirement on the energy $E_n t = 2\pi(n+\gamma)$, it is found that the result is the same as obtained in Ref. 19,

$$\frac{1}{B_n} = \frac{e}{k_0}\left(\frac{\pi \bar{v}_{\text{F}}}{E_{\text{F}}}(n+\gamma) - L\right). \tag{3}$$

Therefore, we found that the key results in the Weyl orbit in type-II Weyl semimetals remain the same as that in type-I Weyl semimetals that was proposed by A.C. Potter, including the quantum oscillation frequency $F_{\text{S}} = \frac{E_{\text{F}} k_0}{e\pi \bar{v}_{\text{F}}}$, thickness dependent quantum oscillations frequency.

Supplementary Note 2. The exclusion of other possible origins of negative MR.

Firstly, the effect of current jetting should be taken into account especially for the point-like contact with sample. Actually, to obtain an Ohmic contact and exclude the effect of residual photoresist on the resistance during nanofabrication, we carefully remove the residual photoresist by Ar plasma before the deposition of electrodes. Moreover, as shown in the microstructure of our sample (Supplementary Figure 1), all the nanoribbons are well surrounded by the Ti (10 nm)/Au (70 nm), instead of a point-like contact.

In the case of current jetting, dips, humps and even the negative voltage will appear in the angular dependence of longitudinal resistance $R(\theta)$.[17,18] To confirm the validity of our data, we

measured $R(\theta)$ of a typical b-axis nanoribbon, as displayed in Supplementary Figure 13. Neither dips nor humps were observed in the $R(\theta)$ curve. This observation confirms that current jetting is not dominated in our nanoribbon samples.

Another evidence to exclude the current jetting is enough large aspect ratio l/w of the sample length l and width w. According to the requirement of homogenous current distribution, the aspect ratio l/w should be larger than $\sqrt{A}$, where A is the resistance anisotropy.[18] The typical resistance anisotropy A in Supplementary Figure 13of is 6.7 ($R(I \perp B)/R_0$=118/17.6=6.7), the aspect ratio l/w is, therefore, required to be higher than $\sqrt{6.7} = 2.6$. In our nanoribbon devices, the width is 0.6-1 μm and the length between two voltage probes (V^+, V^-) is 8-12 μm with a typical aspect ratio l/w ~12. Even though we take A as 57 from the bulk value (Supplementary Figure 13b), the required aspect ratio is $\sqrt{57} = 7.5$, which is still smaller than the real aspect ratio value ~12. Hence, the large aspect ratio l/w in our nanoribbons ensures a homogenous current distribution between two voltage probes.

The resistance maximum related to Knudsen effect normally peaks at maximal boundary scattering of the bent electron trajectories, $2r_c = L$, in which the cyclotron radius is $r_c = \hbar k_F / eB$.[19-21] Therefore, the peak position of magnetic field in the $MR \sim B$ curves will shift towards higher magnetic field with the decrease of nanoribbon thickness (L). Supplementary Figure 14 gives the negative longitudinal magnetoresistance of nanoribbon with various thicknesses, where all the nanoribbons are almost the same in width: 0.6~0.8 μm. We found that the peak position of magnetic field always occurs around 4-5 T without thickness dependence. This observation differs from the expectation from Knudsen effect. Therefore, we can rule out the possibility of Knudsen effect as the origin of negative magnetoresistance.

We found the negative MR is quite sensitive to the thickness. In the semiconducting-like nanoribbon (5.6 nm), only positive MR can be observed. In the nanoribbons with the thickness varying from 12.9 nm to 40.6 nm thick nanoribbons, we could observe the negative MR, see Supplementary Figure 14. In the nanoribbons thicker than 40 nm (Supplementary Figures 14e and 11d), we cannot observe the negative MR anymore. The vanishing of negative MR in thicker ribbons results from the deviation of Fermi level away from the location of Weyl points. The disappearance of negative MR in thinner nanoribbons should be attributed to the vanishing of band crossing between electron and hole pockets, Weyl points, accompanied with the gradual opening of band gap in thinner WTe_2 nanoribbons.[22]

Supplementary Note 3. Thickness dependence of Weyl orbit oscillation phase

According to the theoretical prediction of Weyl orbit quantum oscillation, the dependence of peak position of Weyl orbit on thickness (*L*) is decided by[16]

$$\frac{1}{B_n} = \frac{e}{k_0}\left(\frac{\pi v_{\mathrm{F}}}{E_{\mathrm{F}}}(n+\gamma) - L\right). \tag{4}$$

From Supplementary Eq. (4), the oscillation phase offset of the peaks in the curve ($d^2R/dB^2 \sim B^{-1}$) is expected to be thickness (*L*) dependent although the oscillation period $1/\Delta B$ is independent on thickness. However, it is difficult to identify and extract the precise position (B^{-1}) of Weyl orbit due to the superposition of Weyl orbit and bulk Fermi surfaces in SdH oscillations, because the peaks are close to each other in FFT spectra. Even though we obtain the position of *n*th Landau levels from the inverse FFT spectrum, the large uncertainty of the position will further hinder us from analyzing the oscillation phase on the thickness dependence. This is similar to the case in Moll's work.[21]

Nevertheless, we can expect another kind of oscillation phase shift in the asymmetric Weyl orbit peak in FFT spectrum, arising from the non-adiabatic correction related to the field-induced tunneling between Fermi-arc states and bulk states.[16,21] We analyzed the Weyl orbit peak in FFT spectrum in details. As Supplementary Figure 15 shown, we found that Weyl orbit peak in several curves are quite asymmetric, compared to the peaks of bulk Fermi surfaces in the same spectrum. The asymmetric feature of Weyl orbit is a strong indication of subtle deviation from rigorous periodicity. The shift of peak position B_n towards higher fields will lead to an asymmetric broadening towards low frequency in the FFT spectrum, which is expected for the non-adiabatic correction.[21] The asymmetric feature of Weyl orbit peak in our curves and non-adiabatic corrections are consistent with the observation by Moll et al.[21]

Supplementary Note 4. Examination of Klein tunneling in WTe_2

Recently, O'Brien et al theoretically predict Klein tunneling through a single type-II Weyl point.[23] It is proposed to generate an additional quantum oscillation frequency $|F_+-F_-|$ of two touching Fermi pockets when the magnetic field is perpendicular to the extremal area of Fermi pockets and Fermi level is near the position of potential Weyl points. As discussed in the literatures, this effect is very sensitive to the position of the chemical potential. In single crystal samples of WTe_2, both the first principle calculations and ARPES data indicate that the type II Weyl points are located about 50~80 meV above the chemical potential. Therefore we are not very optimistic to see the Klein tunneling in this particular sample. As shown in Supplementary Figure 16a, in bulk WTe_2 two hole pockets (γ, δ, light blue) and two electron pockets (α, β, purple) are present in the Brillouin zone[8]. We should note that one smaller electron pocket α (and one smaller hole pocket γ) locates inside another larger electron pocket β (and another

larger hole pocket δ), which is similar to a set of Russia dolls.[8] The potential Klein tunneling should occur between the larger electron (β) and larger hole (δ) pockets. According to our experimental 3D mapping of Fermi pockets of WTe_2 (Supplementary Figure 5), the extremal area of Fermi surface in WTe_2 is the *bc* plane. We apply the magnetic field again, therefore, along *a*-axis to check the QO frequency very carefully. As shown in Supplementary Figure 16b, we found that location of four Fermi pockets is consistent with previous result in Supplementary Figure 5. Therefore, the potential additional frequency should be $\left|F_{\mathrm{h}} - F_{\mathrm{e}}\right| = 363(\delta) - 330(\beta) \approx 33\,\mathrm{T}$. However, the data at low frequencies (0~100 T) is too noisy to identify the quantum oscillation due to Klein tunneling. This potential QO frequency $|F_+ - F_-|$ is neither evident nor can be excluded in our experiments. Hence we can't make a clear conclusion based on our observations. Therefore the fact that there is no obvious quantum oscillation at frequency $|F_+ - F_-|$ in our sample is consistent with the previous results from the ARPES measurements and DFT calculations.

Supplementary References

1 Jiang, Y., Gao, J. & Wang, L. Raman fingerprint for semi-metal WTe_2 evolving from bulk to monolayer. *Sci. Rep.* **6**, 19624 (2016).

2 Kim, M. *et al.* Determination of the thickness and orientation of few-layer tungsten ditelluride using polarized Raman spectroscopy. *2D Materials* **3**, 034004 (2016).

3 Song, Q. *et al.* The in-plane anisotropy of WTe_2 investigated by angle-dependent and polarized Raman spectroscopy. *Sci. Rep.* **6**, 29254 (2016).

4 Wang, Y. *et al.* Gate-tunable negative longitudinal magnetoresistance in the predicted type-II Weyl semimetal WTe_2. *Nat. Commun.* **7**, 13142 (2016).

5 Lee, P. A. & Stone, A. D. Universal conductance fluctuations in metals. *Phys. Rev. Lett.* **55**, 1622-1625 (1985).

6 Wang, L. *et al.* Tuning magnetotransport in a compensated semimetal at the atomic scale. *Nat. Commun.* **6**, 8892 (2015).

7 Shoenberg, D. *Magnetic oscillations in metals*. (Cambridge University Press, 2009).

8 Zhu, Z. *et al.* Quantum oscillations, thermoelectric coefficients, and the fermi surface of semimetallic WTe_2. *Phys. Rev. Lett.* **114**, 176601 (2015).

9 Huang, X. *et al.* Observation of the chiral-anomaly-induced negative magnetoresistance in 3D Weyl semimetal TaAs. *Phys. Rev. X* **5**, 031023 (2015).

10 Kim, H.-J. *et al.* Dirac versus Weyl fermions in topological insulators: Adler-Bell-Jackiw anomaly in transport phenomena. *Phys. Rev. Lett.* **111**, 246603 (2013).

11 Tchoumakov, S., Civelli, M. & Goerbig, M. O. Magnetic-Field-Induced Relativistic Properties in Type-I and Type-II Weyl Semimetals. *Phys. Rev. Lett.* **117**, 086402 (2016).

12 Udagawa, M. & Bergholtz, E. J. Field-Selective Anomaly and Chiral Mode Reversal in Type-II Weyl Materials. *Phys. Rev. Lett.* **117**, 086401 (2016).

13 Yu, Z. M., Yao, Y. G. & Yang, S. Y. A. Predicted Unusual Magnetoresponse in Type-II Weyl Semimetals. *Phys. Rev. Lett.* **117**, 077202, (2016).

14 Soluyanov, A. A. *et al.* Type-II Weyl semimetals. *Nature* **527**, 495-498 (2015).

15 Wang, C. *et al.* Observation of Fermi arc and its connection with bulk states in the candidate type-II Weyl semimetal WTe_2. *Phys. Rev. B* **94**, 241119 (2016).

16 Potter, A. C., Kimchi, I. & Vishwanath, A. Quantum oscillations from surface Fermi arcs in Weyl and Dirac semimetals. *Nat. Commun.* **5**, 5161 (2014).

17 Arnold, F. *et al.* Negative magnetoresistance without well-defined chirality in the Weyl semimetal TaP. *Nat. Commun.* **7**, 11615 (2016).

18 Dos Reis, R. *et al.* On the search for the chiral anomaly in Weyl semimetals: The negative longitudinal magnetoresistance. *New J. Phys.* **18**, 085006 (2016).

19 Thornton, T., Roukes, M., Scherer, A. & Van de Gaag, B. Boundary scattering in quantum wires. *Phys. Rev. Lett.* **63**, 2128-2131 (1989).

20 De Jong, M. & Molenkamp, L. Hydrodynamic electron flow in high-mobility wires. *Phys. Rev. B* **51**, 13389-13402 (1995).

21 Moll, P. J. *et al.* Transport evidence for Fermi-arc-mediated chirality transfer in the Dirac semimetal Cd_3As_2. *Nature* **535**, 266-270 (2016).

22 Tang, S. *et al.* Quantum spin Hall state in monolayer 1*T*-WTe_2. *Nat. Phys.* **13**, 683-687 (2017).

23 O'Brien, T., Diez, M. & Beenakker, C. Magnetic breakdown and Klein tunneling in a type-II Weyl semimetal. *Phys. Rev. Lett.* **116**, 236401 (2016).
