# Supplementary material for: Evidence for topological type-II Weyl semimetal WTe2
Source: Nat Commun. 2017 Dec 15;8:2150. doi: 10.1038/s41467-017-02237-1 (PMC5732285; doi:10.1038/s41467-017-02237-1)
Supplement: Supplementary file 2 — Peer Review File [file 41467_2017_2237_MOESM2_ESM.pdf]

Editorial Note: Parts of this peer review file have been redacted as indicated to maintain the confidentiality of unpublished data.

Reviewers' comments:

Reviewer #1 (Remarks to the Author):

In this well written and impressive work the authors present evidence for WTe2 being a type-II Weyl semimetal from studies of magnetotransport in nanoribbons of this material. Two key findings are presented: (1) a new magnetic quantum oscillation (MQO) frequency in the Shubnikov de Haas frequency spectrum and (2) a negative magnetoresistance occurring when the field is applied parallel to the current. I think the first finding by itself makes this work worth publishing in a high-rank journal such as Nat Comm. The second result, however, needs to be handled extremely carefully. Altogether, I would suggest this paper to be considered for an article in Nature Communication after the authors address a couple of my comments below.

First, I think the overall study appears to be carried out very carefully and comprehensively. The analysis of the MQOs seems solid and the 2D nature of the new oscillation (~78T) is clearly observed and supports the presence of surface states for this compound. In addition, that the oscillation vanishes as the thickness increases is what is expected for such an exotic state. I would ask for a few further details about the technical part, e.g. contact resistances and currents used for the MR measurements, to make sure overheating was no issue. Furthermore, I would ask to add error bars to the frequency and effective mass values determined from fitting only few temperatures (with a gap between 10 and 20 K). In addition, it would be interesting to learn for how many samples the 78T and its effective mass has been reproduced.
One unique feature for the proposed surface state MQO would be a strong dependence of the oscillation phase depending on the thickness of the ribbons, see reference 12 (Moll et al. 2016). Could the authors comment on that with respect to their findings?

Second, I think the authors need to convince me and likely others that current jetting and the Knudsen effect can be ruled out as the origin of the observed negative MR! Negative longitudinal MR is somewhat ambiguous and has been reported for various materials. In particular compensated (high-mobility) semi-metals (see for example Steele et al.,Phys. Rev. 97, 1720 (1955) or Reis at al., New J. Phys. 18 (2016) 085006) are known for field induced anisotropies and current jetting effects that may lead to such anomalous transport signals. So do the investigated ribbons fulfill the dimension requirement for a homogeneous current distribution for such a strong inplane mobility anisotropy?
From my point of view the fact that the nMR is seen solely for single direction and within a tiny angular range speaks for a non-trivial origin. How many samples were measured to reproduce this result? I wonder how this effect changes with the thickness and width of the samples.

Abstract:
“Electron and hole pockets” may be better understandable by adding Fermi surface or band in front of pockets

Page 2:
13 oblique conduction and the valence pockets (not clear?) using bands instead of pockets may be better here
18 ARPES is one possible technique (not the only one) to observe the surface states directly; are the arcs so much smaller than in MoTe2, where ARPES was able to observe them.
22 connecting Fermi arcs “via” bulk WPs

Page 3:
1 chiral anomaly … introduce properly what is meant by anomaly?
2 lead to an extra quantum oscillation frequency in MR measurements … why only MR? --> it should lead to an additional frequency in the MQOs spectra for experiments that probe the FS, such as magnetotransport measurements.

3 neg MR has been reported for many other WSMs but extreme care must be taken to distinguish it from the current jetting effect in high-mobility semimetals!--> comment on that please
17 predication --> prediction

Page 5:
6-7 peaks are shifted by how much? Better to say different frequencies correspond to different Fermi surface sizes, i.e. shift in the chemical potential

Page 11:
How is the negMR affected by the various thicknesses?

Page12:
Applied currents? Contact resistances for the various samples investigated?

Page 14:
As demonstrated previously --> As predicted 19 and demonstrated 12 previously…

Conclusion:

Would the authors propose any other experiments that could be revealing besides ARPES? Furthermore, how do the authors rate the impact of their work on further research that may be excited by their findings?

Supplementary Info:

S4 What is the error bar for the effective masses?

How did the R versus T for the FIB prepared sample compare to bulk samples? What contact resistance did they have? Did the resistivity match?

Furthermore, the exotic Weyl nature of the observed surface state could be distinguished from more conventional surface states by study of their presence even in case the surface of the sample was destroyed. Here, the FIB may be the right tool to perform such a test. Unlike a conventional surface state, a Weyl state would still be detectable and may become even stronger pronounced after a sensible ion exposure, as the bulk is becoming smaller and hence the distance between the arcs too.

Reviewer #2 (Remarks to the Author):

The current paper deals with a candidate material for a type II Weyl semimetal, WTe2. This is an interesting choice of topic as type II WSM are relatively new and so far are a theoretical idea only. Therefore, finding a realization of this theoretical systems is a considerable breakthrough.

The authors of the paper perform transport measurements in order to determine whether WTe_2 is indeed a type II WSM. They base their affirmative determination on two observables - Magnetoresistence and Quantum oscillations.

It is important to mention here that while type-I WSM have only Fermi points in the bulk and Fermi arcs on some surfaces, type II WSM have non-zero density of states at the Weyl point energy. At this energy, the bulk Fermi surface contains an electron and hole pocket and the low energy spectrum is not gapped. For this reason, any surface arc state is not protected against scattering into the bulk by disorder. This is the main difference between the two types of WSMs

and it is the Fermi pockets that experiments should be after.

The current manuscript bases their quantum oscillations analysis on Refs. 12 and 19, both of them written before the discovery of type II Weyl semimetals. In fact, the 'Weyl orbit' which produces the oscillations which involve the Fermi arcs should be modified or doesn't even exist in a type II WSM.
For this reason I find the QO discussion confusing and I am not convinced that the data really supports the type II WSM hypothesis. Moreover, Ref. 19 predicts a thickness-dependent quantum oscillations frequency and the authors do not check for thickness.

I suggest to the authors to analyze the unique signatures of *bulk* quantum oscillations as proposed by O'Brien et al. in PRL 116, 236401. They propose that magnetic breakdown appears very distinctly.

1. Following the comments of the referee, the current jetting and Knudsen effect were carefully analyzed and discussed to demonstrate that they are not the origin of the observed negative magnetoresistance. The detailed analyses were added into text and SI note 1. Please see line 22 page 10 in main text.

2. More measurements have been performed on different samples and more experimental data have been collected on Weyl orbit quantum oscillation and negative MR data. These data were analyzed and added in the main text and SI. Please find in the updated Figure 4, Figures S6 and S7, and Figure S13.

3. The error bars of effective mass were added. Please see updated Figures S4, S6, S7, line 19 page 5, and line 14 page 8 in main text.

4. We carefully checked the quantum oscillations to detect the possible Klein tunneling in our sample. The detailed discussion was added into SI note 3. Please see line 15 page 16 in main text.

5. The reason that we can't extract the thickness dependence of Weyl orbit oscillation phase is analyzed. Please see SI note 2.

6. All the revisions were highlighted in the text by red color words.

Response to Reviewer #1 :

Reviewer #1: In this well written and impressive work the authors present evidence for WTe2 being a type-II Weyl semimetal from studies of magnetotransport in nanoribbons of this material. Two key findings are presented: (1) a new magnetic quantum oscillation (MQO) frequency in the Shubnikov de Haas frequency spectrum and (2) a negative magnetoresistance occurring when the field is applied parallel to the current. I think the first finding by itself makes this work worth publishing in a high-rank journal such as Nat Comm. The second result, however, needs to be handled extremely carefully. Altogether, I would suggest this paper to be considered for an article in Nature Communication after the authors address a couple of my comments below.

Comment 1: *First, I think the overall study appears to be carried out very carefully and comprehensively. The analysis of the MQOs seems solid and the 2D nature of the new oscillation (~78T) is clearly observed and supports the presence of surface states for this compound. In addition, that the oscillation vanishes as the thickness increases is what is expected for such an exotic state. I would ask for a few further details about the technical part, e.g. contact resistances and currents used for the MR measurements, to make sure overheating was no issue. Furthermore, I would ask to add error bars to the frequency and effective mass values determined from fitting only few temperatures (with a gap between 10 and 20 K). In addition, it would be interesting to learn for how many samples the 78T and its effective mass has been reproduced.*

Reply: Thank you for your positive comments. To extract the contact resistance, we compare the resistance of the typical nanoribbon through two-probe and four-probe methods, as shown in Fig. R1-1a. Both current-voltage curves show excellent linear behaviors, suggesting a very good Ohmic contact. A very small resistance difference of ~10 Ω is found. Compared to the

standard four-probe method, this small resistance difference should contain the contact resistance and the electrode resistance of two voltage probes (Ti(10 nm)/Au(70 nm)). We believe the contact resistance in our Ohmic-contacted samples is negligible, compared to the semimetallic WTe_2. Otherwise, it is really difficult to observe the high-quality quantum oscillations in poor-contacted samples. Figure R1-2 gives a representative data in the device without removing the residual photoresists before the Ti/Au deposition. The SdH oscillation in Fig. R1-2b is nearly indistinguishable although we can notice the high-field oscillatory behavior under the linear background in Fig. R1-2a. To avoid heating or damaging nanoribbons by high current density, an AC current of 1 μA is applied during our lock-in measurement.

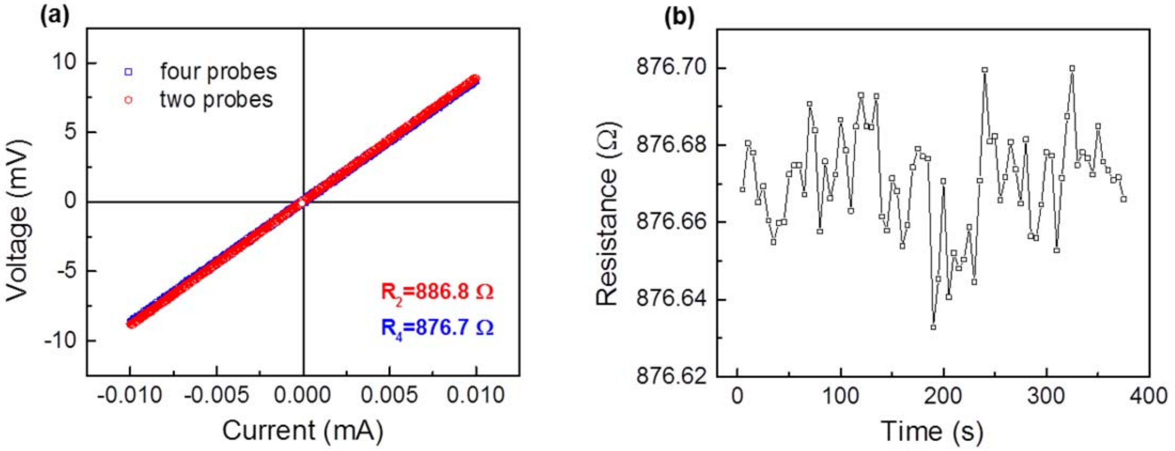

Figure R1-1 (a) typical current-voltage curves with different methods, (b) time dependent resistance that is measured through four probes method on the sample.

To estimate the error bar, we carefully check the resistance variation in our samples, since the frequency and effective mass were analyzed from the resistance. We measured the time dependent resistance at 2 K, as shown in Fig. R1-1b. This observation further excludes the problem of overheating by contact resistance. If the overheating is serious in our nanoribbons, we should expect a gradual increase of resistance in semimetallic WTe_2 as the time increases. The resistance variation in Fig. R1-1b is, nevertheless, random and small. Therefore, we use

the standard instrument error 5% to estimate the frequency and effective mass error bar. The standard error bar for frequency has been added into main text (line 19 page 5, and line 14 page 8), Fig. S4b-4d, and Figs. S6 and S7.

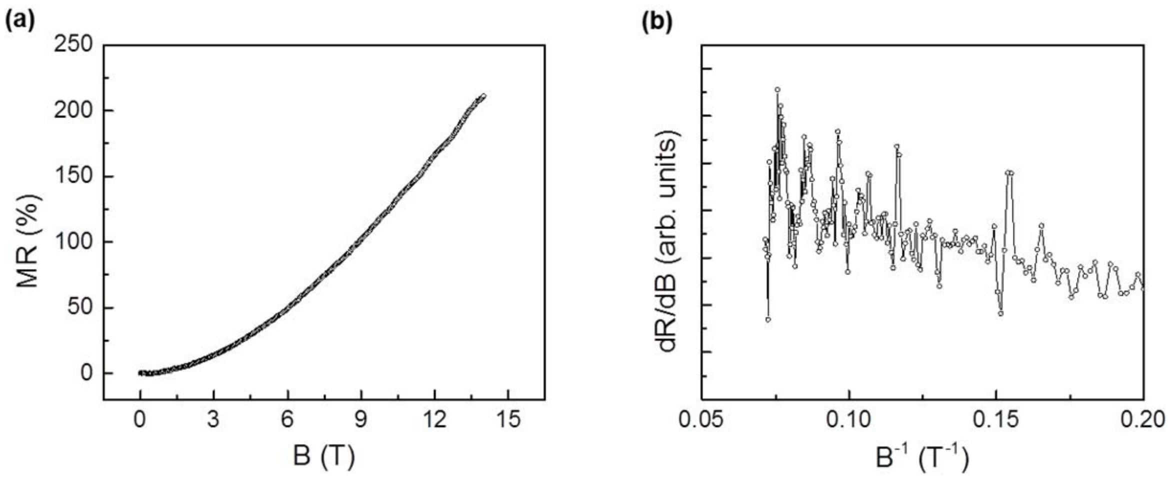

Figure R1-2 typical data of a nanoribbon device without removing the residual photoresist before the deposition of Ti/Au electrodes. (a) field dependent MR at 2 K, (b) noisy SdH oscillation.

To further confirm our results on the effective mass, we measured the temperature dependent quantum oscillations in two more devices with the temperature step of 1 K from 2 K to 10 K, then 12 K, 15 K, and 20 K, which allow us to extract the information based on more data. Therefore, we have extracted very similar values of the effective mass in total 3 samples and observed the existence of the new frequency of ~78.0 T in total 6 samples with various thicknesses. Figures R1-3 and R1-4 show the data for extracting the effective mass and new Weyl orbit frequency in two nanoribbons with thickness of 35.0 nm and 16.8 nm. Therefore, we can assure that the effective mass value and new Weyl orbital frequency of ~78.0 T±3.9 T are valid and reproducible. Based on the new results, we have then updated Fig. 4 Figs. S6 and S7.

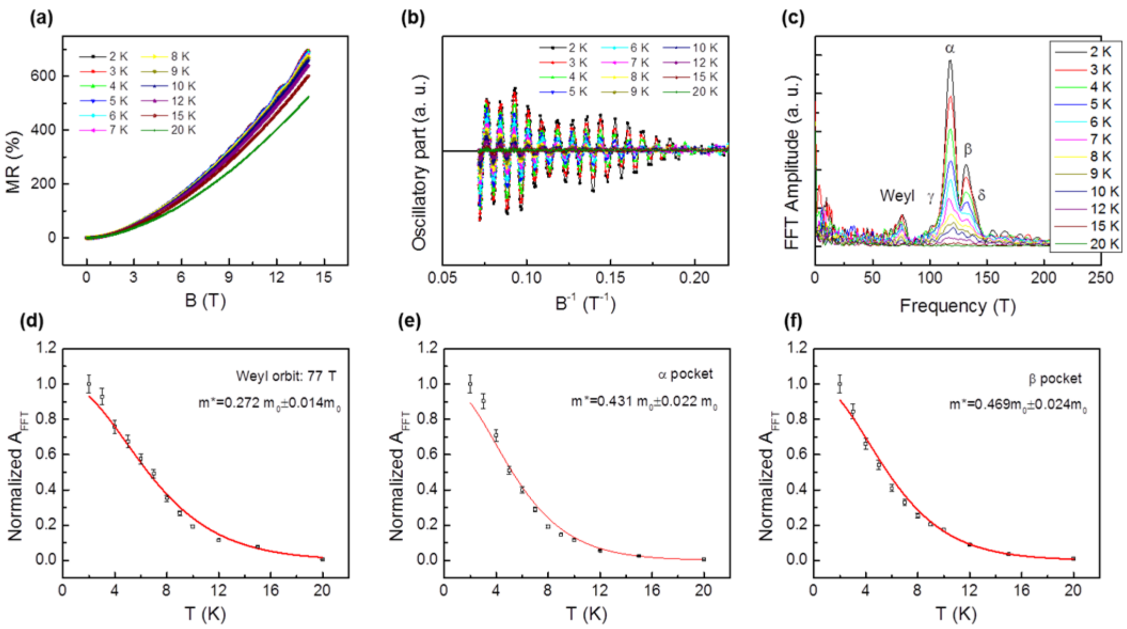

Figure R1-3 Reproducible data of a *b*-axis nanoribbon with thickness *t*=35.0 nm for obtaining new Weyl orbit frequency and effective mass. (a) Field dependent MR at various temperatures, (b) SdH oscillations in the plots of $d^2R/dB^2 \sim B^{-1}$, (c) The FFT spectra at various temperatures. The temperature dependence of normalized FFT amplitude and fitting of effective mass. (d) Weyl orbit, (e) α pocket, (f) β pocket.

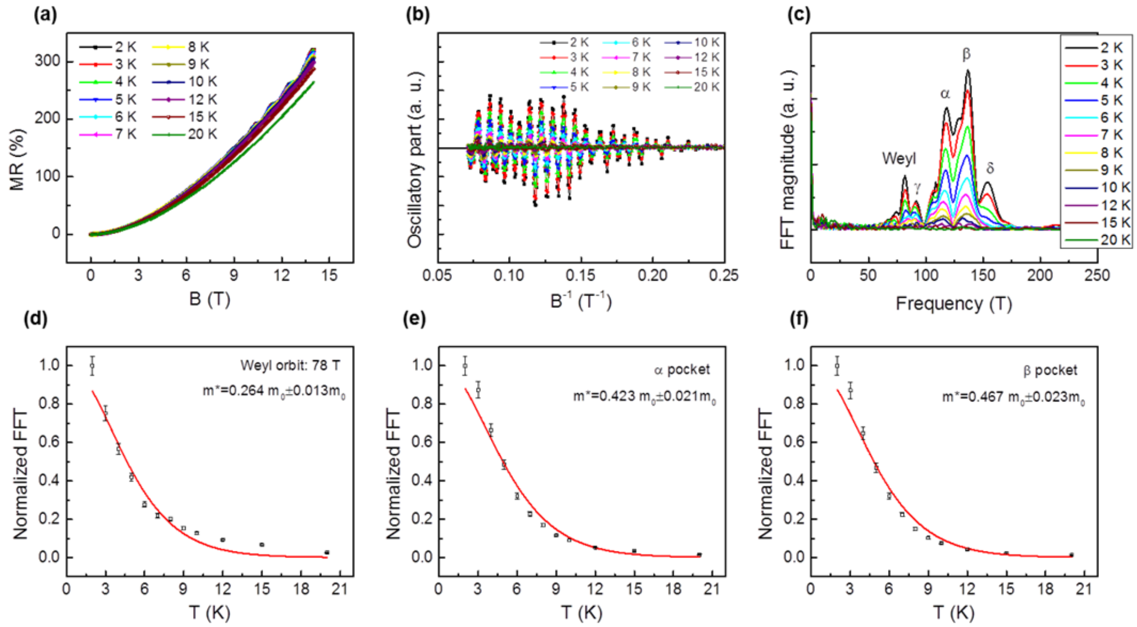

Figure R1-4 Reproducible data of a *b*-axis nanoribbon with thickness *t*=16.8 nm for obtaining new Weyl orbit frequency and effective mass. (a) Field dependent MR at various temperatures, (b) SdH oscillations in the plots of $d^2R/dB^2 \sim B^{-1}$, (c) The FFT spectra at various temperatures. The temperature dependence of normalized FFT amplitude and fitting of effective mass. (d) Weyl orbit, (e) α pocket, (f) β pocket.

***Comment 1-1:** One unique feature for the proposed surface state MQO would be a strong dependence of the oscillation phase depending on the thickness of the ribbons, see reference 12 (Moll et al. 2016). Could the authors comment on that with respect to their findings?*

Reply: Yes, the oscillation phase offset of the peaks in the curve ($d^2R/dB^2 - B^{-1}$) is expected to be thickness dependent although the oscillation period $1/\Delta B$ is independent on thickness. According to the theoretical prediction of Ref. 19, the dependence of peak position of Weyl orbit on thickness (*L*) is decided by[1]

$$\frac{1}{B_n} = \frac{e}{k_0}\left(\frac{\pi v_F}{E_F}(n+\gamma) - L\right). \qquad \text{(R1)}$$

However, it is difficult to identify and extract the precise position (B^{-1}) of Weyl orbit due to the superposition of Weyl orbit and bulk Fermi surfaces in SdH oscillations and the peaks close to each other in FFT spectra. Even though we obtained the position of *n*th Landau levels from the inverse FFT spectrum, the large uncertainty of the position will further hinder us from analyzing the oscillation phase on the thickness dependence. This is similar to the case in Moll's work.

Nevertheless, we would expect to observe another kind of oscillation phase shift of the asymmetric Weyl orbit peak in FFT spectrum, which should arise from the non-adiabatic correction related to the field-induced tunneling between Fermi-arc states and bulk states.[1,2] We analyzed the Weyl orbit peak in FFT spectrum in details. As shown in Fig. R1-5, the Weyl orbit peak in several curves, compared to the peaks of bulk Fermi surfaces in the same spectrum, is quite asymmetric. The asymmetric feature of Weyl orbit is a strong indication of deviation from strict periodicity. The shift of peak position B_n towards higher fields will lead to an asymmetric broadening towards low frequency in the FFT spectrum, which should be ascribed to the non-adiabatic correction[1,2]. Therefore, the asymmetric feature of Weyl orbit peak observed in these

studies has the origin of non-adiabatic corrections, which is consistent with the observation by Moll et al.[2] The analysis on Weyl orbit oscillation phase has been added into SI note 2.

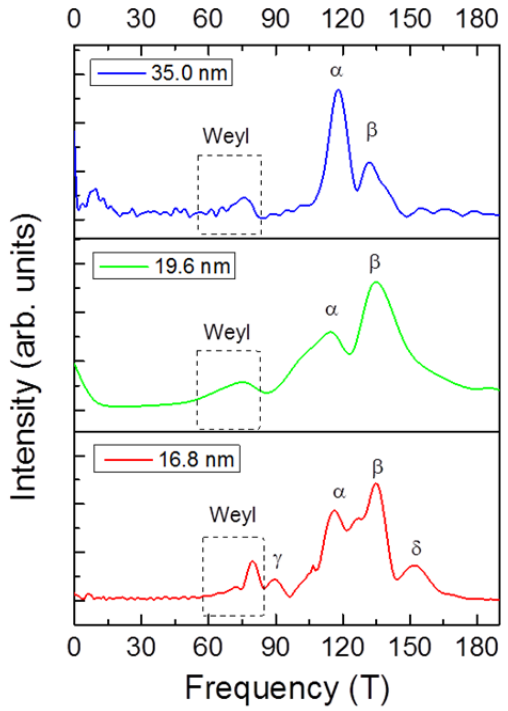

Figure R1-5 Asymmetric Weyl orbit peak in FFT spectra of nanoribbons with different thicknesses. The peaks in the dashed rectangles show asymmetric broadening towards low frequency due to the non-adiabatic correction.

Comment 2: *Second, I think the authors need to convince me and likely others that current jetting and the Knudsen effect can be ruled out as the origin of the observed negative MR! Negative longitudinal MR is somewhat ambiguous and has been reported for various materials. In particular compensated (high-mobility) semi-metals (see for example Steele et al.,Phys. Rev. 97, 1720 (1955) or Reis at al., New J. Phys. 18 (2016) 085006) are known for field induced anisotropies and current jetting effects that may lead to such anomalous transport signals. So do the investigated ribbons fulfill the dimension requirement for a homogeneous current distribution for such a strong in plane mobility anisotropy?*

Reply: Thank you for your suggestion on excluding the other possible origins of negative magnetoresistance.

First, we fully agree with you that the effect of current jetting should be taken into account especially for the point-like contact to the samples. Actually in our measurements of longitudinal MR, all the nanoribbons are well surrounded by the Ti (10 nm)/Au (70 nm) with excellent Ohmic contact (Fig. R1-1), instead of a point-like contact, as shown in the microstructure of our sample (Fig. S1 and inset of Fig. 3d). Moreover, to avoid the problems of "local variation of the contact resistance of the electrode that may lead to an injection of the current into the sample where the contact resistance is smallest, as reported by Reis et al for the silver paint contacts.[3] We have made the contacts very carefully. To obtain an Ohmic contact and exclude the effect of residual photoresist during nanofabrication, we carefully remove the residual photoresist by Ar plasma before the deposition of electrodes.

In the case of current jetting, dips, humps and even the negative voltage will appear in the angular dependence of longitudinal resistance R(θ).[3,4] To confirm the validity of our data, we measured R(θ) of a typical *b*-axis nanoribbon, as displayed in Fig. R1-6. Neither dips nor humps were observed in the R(θ) curve, which is sharp contrast to what reported by Reis at al.[3] This observation confirms that current jetting is not dominated in our nanoribbon samples.

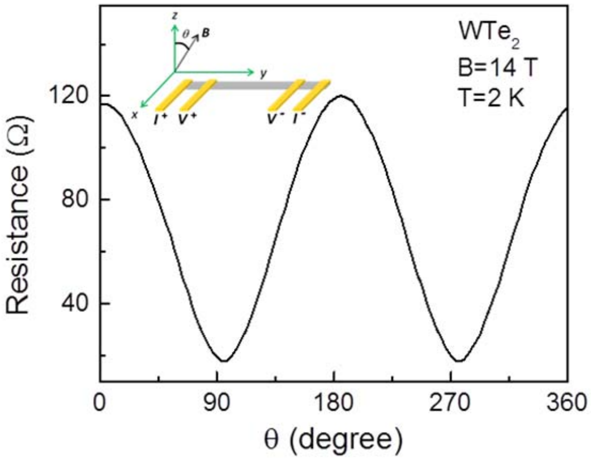

Fig. R1-6 Typical angular dependence of longitudinal resistance of *b*-axis nanoribbon under a magnetic field of 14 T and 2 K. The inset gives the measurement configuration.

Another evidence to exclude the current jetting is enough large aspect ratio *l*/*w* of the sample length *l* and width *w*. According to the requirement of homogenous current distribution, the aspect ratio *l*/*w* should be larger than $\sqrt{A}$, where *A* is the resistance anisotropy.[3] The typical resistance anisotropy *A* in Fig. R1-6 of is 6.7 ($R(I \perp B)/R_0$=118/17.6=6.7), the aspect ratio *l*/*w* is, therefore, required to be higher than $\sqrt{6.7} = 2.6$. In our nanoribbon devices, the width is 0.6-1 μm and the length between two voltage probes (V^+, V^-) is 8-12 μm with a typical aspect ratio *l*/*w* ~12. Even though we take *A* as 57 from the bulk value (Fig. S3b), the required aspect ratio is $\sqrt{57} = 7.5$, which is still smaller than the real aspect ratio value ~12. Hence, the large aspect ratio *l*/*w* in our nanoribbons ensures a homogenous current distribution between two voltage probes.

Another sharp difference between our longitudinal MR and the one reported by Reis at al, is the magnitude or the size of the MR. The values of MR (positive peak and negative MR for both deduced from V1 or V2) are much larger than we observed here (at least one order of

magnitude larger) in the field range up to 14 T.[3] Therefore, our data can't be ascribed to the effect of current jetting.

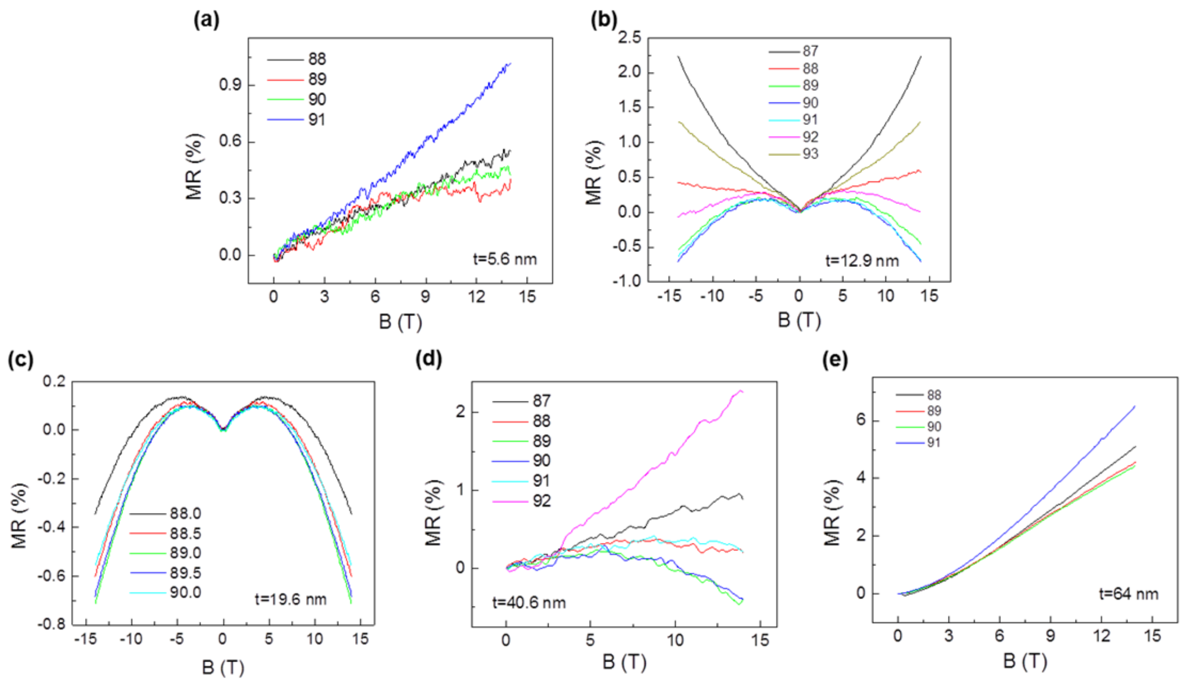

Fig. R1-7 Chiral anomaly induced negative magnetoresistance in the nanoribbons with various thicknesses. All the data were obtained at 2 K. The disappearance of negative MR in thinner (a) and thicker nanoribbons (e) will be discussed in the reply to next comment.

The resistance maximum related to Knudsen effect normally peaks at maximal boundary scattering of the bent electron trajectories, $2r_c = L$, in which the cyclotron radius is $r_c = \hbar k_F / eB$.[2,5,6] Therefore, the peak position of magnetic field in the $\mathrm{MR} - B$ curves will shift towards higher magnetic field with the decrease of nanoribbon thickness (*L*). Figure R1-7 gives the negative longitudinal magnetoresistance of nanoribbon with various thicknesses, where all the nanoribbons are almost the same in width: 0.6~0.8 μm. We found that the peak position of magnetic field always occurs around 4~5 T without thickness dependence. This observation differs from the expectation from Knudsen effect. Therefore, we can rule out the possibility of

Knudsen effect as the origin of negative magnetoresistance. The disappearance of negative MR in thinner and thicker nanoribbons (Fig. 1-7a&e) will be discussed in the reply to next comment.

The discussion on the exclusion of other origins of negative MR was added into main text and SI note 1. Please see line 22 page 10 in main text.

Comment 2-1: *From my point of view the fact that the nMR is seen solely for single direction and within a tiny angular range speaks for a non-trivial origin. How many samples were measured to reproduce this result? I wonder how this effect changes with the thickness and width of the samples.*

Reply: As you commented, the negative MR that has the non-trivial origin only exists for a tiny angular range (89°~92°), such as the data in Fig. R1-7b. In our observations, the chiral anomaly induced negative MR shows no significant dependence on the width of nanoribbons. We can obtain the negative MR in the *b*-axis nanoribbons with width ranging from ~120 nm to ~2 um (with the same thickness ~20 nm), as shown in Fig. R1-8. Although the universal conductance fluctuation is obvious in 120 nm wide nanoribbon, the negative MR is still distinguishable (Fig. S2 or Fig. R1-8a).

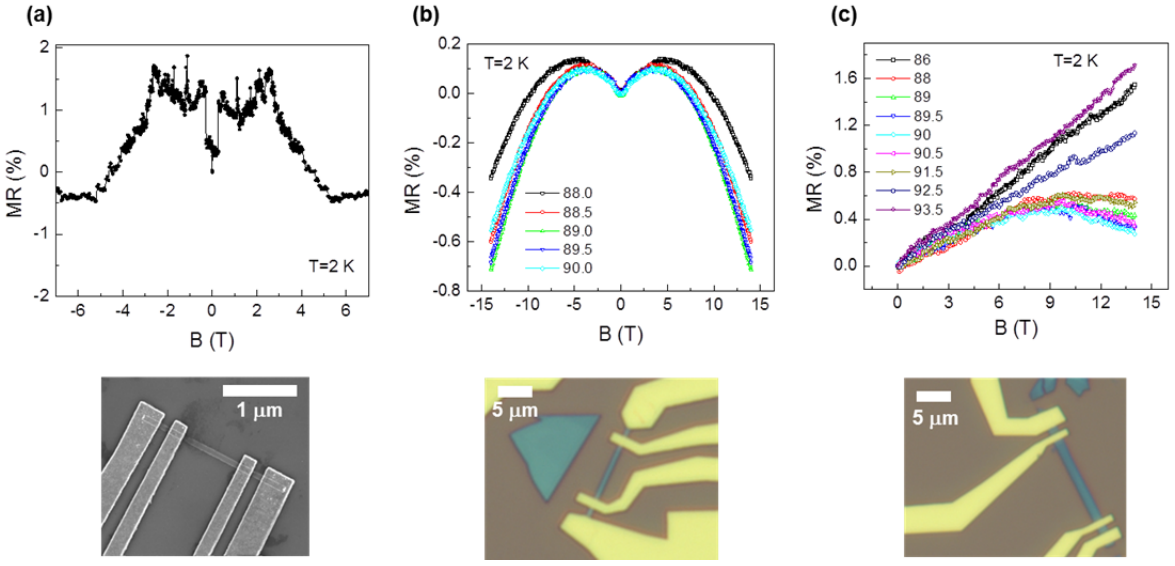

Fig. R1-8 Negative magnetoresistance of *b*-axis nanoribbons (*t*=20 nm) with different width (*w*). The upper panel gives the field dependent magnetoresistance, and the bottom panel shows the corresponding images. (a) *w*=120 nm, (b) *w*=650 nm, (c) *w*=2 um.

On the contrary, the negative MR is more sensitive to the thickness. In the semiconducting-like nanoribbon (5.6 nm), only positive MR can be observed. In the nanoribbons with the thickness varying from 12.9 nm to 40.6 nm thick nanoribbons, we could observe the negative MR, see Fig. R1-7. In the nanoribbons thicker than 40 nm (Fig. R1-7f and Fig. S11d), we cannot observe the negative MR anymore. The vanishing of negative MR in thicker ribbons results from the deviation of Fermi level away from the location of Weyl points. The disappearance of negative MR in thinner nanoribbons should be attributed to the vanishing of band crossing between electron and hole pockets, Weyl points, accompanied with the gradual opening of band gap in thinner WTe_2 nanoribbons.[7]

As to the number of sample with negative MR, we believe that we can repeat the negative MR in more *b*-axis nanoribbons with the thickness varying from ~10 nm to ~40 nm, although we only repeated the observation in 5 *b*-axis nanoribbons (Fig. R1-7 and R1-8).

Several Detailed Comments:

Main Text parts:

1. Abstract: "Electron and hole pockets" may be better understandable by adding Fermi surface or band in front of pockets.

Reply: Thanks for your suggestion. "Fermi surface" has been added in front of pockets.

2. Page 2:

13, oblique conduction and the valence pockets (not clear?) using bands instead of pockets may be better here;

18, ARPES is one possible technique (not the only one) to observe the surface states directly; are the arcs so much smaller than in MoTe2, where ARPES was able to observe them.

22, connecting Fermi arcs "via" bulk WPs.

Reply: 13, we substitute bands for pockets.

18, Thank you for your reminder. The methods including ARPES and scanning tunneling spectroscopy are possible technique to observe the surface states directly. We add scanning tunneling spectroscopy into the text. Yes, the calculated Fermi arc in WTe_2 is 0.032 $Å^{-1}$, much smaller than that of $MoTe_2$ (0.081 $Å^{-1}$).[8-12] The direct observation the Fermi arc in $MoTe_2$ has been realized in several works.[9,10,12]

22, we substitute via for with.

3. Page 3:

1, chiral anomaly … introduce properly what is meant by anomaly?

2, lead to an extra quantum oscillation frequency in MR measurements … why only MR? --> it should lead to an additional frequency in the MQOs spectra for experiments that probe the FS, such as magnetotransport measurements.

3, neg MR has been reported for many other WSMs but extreme care must be taken to distinguish it from the current jetting effect in high-mobility semimetals!--> comment on that please

17, predication --> prediction

Reply: 1. We define chiral anomaly in text as following. Chiral anomaly refers to the state that the particle number with given chirality is non-conserved when a magnetic field is parallel to the direction of electric field. Please see line 6 page 3.

2. We corrected the expression according to your good suggestion.

3. Please find the detailed replies in comment 2. We have added the detailed discussion on current jetting and Knudsen effect into the supplementary information.

17. The typo was corrected.

__4. Page 5:__

6-7 peaks are shifted by how much? Better to say different frequencies correspond to different Fermi surface sizes, i.e. shift in the chemical potential

Reply: The peaks are shifted by 2 T(γ), 8 T(α), 7 T(β), 5 T(δ), respectively. We changed the expression by following your suggestion. Please see line 7 page 5 in main text.

__5. Page 11:__

How is the negMR affected by the various thicknesses?

Reply: Please find the detailed discussion in Reply to comment 2-1. The negative MR can be observed in the *b*-axis nanoribbons with thickness ranging from ~10 nm to ~40 nm. We added this part into the SI note 1.

__6. Page12:__

Applied currents? Contact resistances for the various samples investigated?

Reply: A current of 1 μA is applied during the measurements. The contact resistance should be smaller than 10 Ω. See detailed analysis in reply to comment 1.

__7. Page 14:__

As demonstrated previously --> As predicted 19 and demonstrated 12 previously…

Reply: “predicted” and reference were added.

8. Conclusion:

Would the authors propose any other experiments that could be revealing besides ARPES? Furthermore, how do the authors rate the impact of their work on further research that may be excited by their findings?

Reply: For instance, scanning tunneling spectroscopy is another powerful technique to reveal band structures and surface states. The angle-resolved photoemission spectroscopies with different light sources, such as laser, soft x-ray and synchrotron, are also powerful tools to detect.

We think our work reveal the experimental realization of the first predicted type-II Weyl fermions in condensed matters. And it should be the first transport demonstration on the existence of type-II Weyl fermions and Fermi arcs.

Our work and the work in Dirac semimetal Cd_3As_2, together with the theoretical prediction, have demonstrated that the magneto-transport measurement is a reliable and convenient technique to demonstrate the existence of topological Fermi arcs in Weyl and Dirac semimetals.

We have added this part into summary part. Please see line 1 page 17 in main text.

Supplementary parts:

9. S4 What is the error bar for the effective masses?

Reply: According to your suggestion, the error bar and uncertainty is also added in Fig. S4 and text. Please see line 19 page 5, and line 14 page 8 in main text.

10. How did the R versus T for the FIB prepared sample compare to bulk samples? What contact resistance did they have? Did the resistivity match?

Reply: The temperature dependent resistivity of FIB sample is in between bulk sample and several layered nanoribbons, as the comparison shown in Fig. R1-9a. The contact resistance, which we roughly estimated from the resistance difference in between the two-probes method and four-probes method, is smaller than 1.5 Ω. The current-voltage curves in Fig. R1-9b are quite linear, indicating an Ohmic contact between the FIB Pt and WTe_2. The resistivity of Pt and WTe_2 matches well.

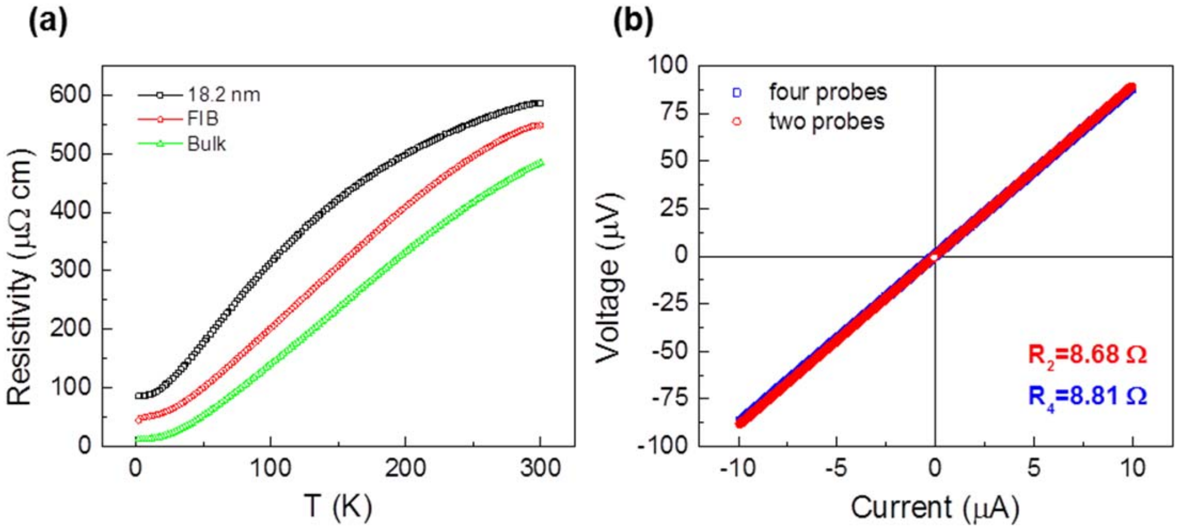

Fig. R1-9 (a) Comparison of temperature dependent resistivity of WTe_2. (b) Current-voltage curves of FIB sample that are measured with two probes and four probes.

11. Furthermore, the exotic Weyl nature of the observed surface state could be distinguished from more conventional surface states by study of their presence even in case the surface of the sample was destroyed. Here, the FIB may be the right tool to perform such a test. Unlike a conventional surface state, a Weyl state would still be detectable and may become even stronger pronounced after a sensible ion exposure, as the bulk is becoming smaller and hence the distance between the arcs too.

Reply: Thanks for your suggestion the ion exposure. The Weyl state is indeed expected to be even stronger after the ion exposure, such as the observation in the Cd_3As_2 slab.[2] To check this effect, we alter the natural exfoliated (001) surface of WTe_2 nanoribbon (t=16.8 nm) through very gentle Ga^+ ion exposure, where the exposure condition is V=5 kV, I=43 pA and t=3 s. However, it can be seen in Fig. R1-10 that the conduction mechanism of exposed nanoribbon becomes semiconducting from metallic behavior. Meanwhile, the MR is largely suppressed and SdH oscillation nearly disappears. Unexpectedly, the Weyl orbit vanishes after the ion exposure. The data before ion exposure were shown in Fig. R1-4. There are probably two reasons to account for this phenomenon. The first is that we didn't have the enough experience to find the suitable FIB condition in which we alter the surface only but keep the intrinsic physical properties unchanged. On the other hand, the Weyl points exists only on the WTe_2 (001) surface owing to the reflection symmetry,[8] which is different from the 3D Dirac point and Fermi arc along $\Gamma - Z$ direction in Cd_3As_2.[13] The Weyl orbit vanished maybe due to destroy of WTe_2 (001) top surface.

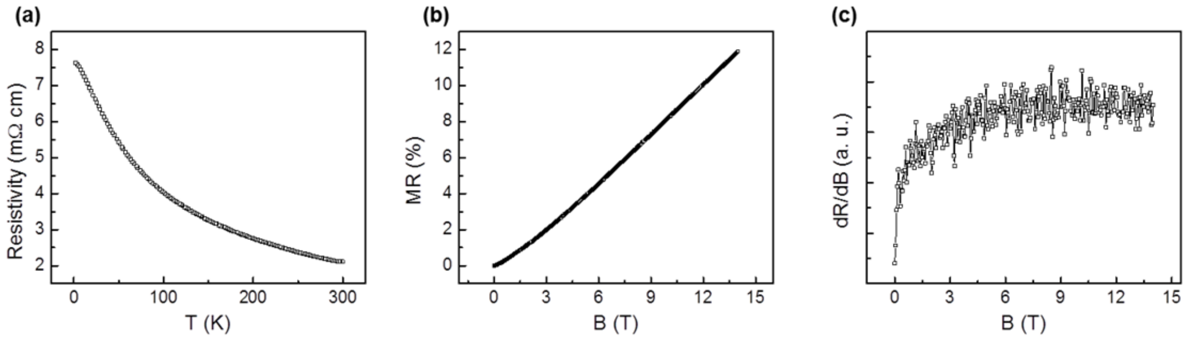

Fig. R1-10 Magneto-transport property of *b*-axis WTe_2 ribbon (*t*=16.8 nm) that is exposure by Ga+ in FIB. (a) Temperature dependence of resistivity, (b) field dependence of magnetoresistance measured at 2 K with *B*//*c*, (c) field dependence of first derivative of resistance in (b).

Response to Reviewer #2 :

Reviewer #2:

The current paper deals with a candidate material for a type II Weyl semimetal, WTe2. This is an interesting choice of topic as type II WSM are relatively new and so far are a theoretical idea only. Therefore, finding a realization of this theoretical systems is a considerable breakthrough.

The authors of the paper perform transport measurements in order to determine whether WTe_2 is indeed a type II WSM. They base their affirmative determination on two observables - Magnetoresistence and Quantum oscillations.

It is important to mention here that while type-I WSM have only Fermi points in the bulk and Fermi arcs on some surfaces, type II WSM have non-zero density of states at the Weyl point energy. At this energy, the bulk Fermi surface contains an electron and hole pocket and the low energy spectrum is not gapped. For this reason, any surface arc state is not protected against scattering into the bulk by disorder. This is the main difference between the two types of WSMs and it is the Fermi pockets that experiments should be after.

Reply: Thank you for your comments on the nature of surface arc states. We understand that the key issue you pointed out is the topology nature of surface arc state in type-II Weyl semimetals. Indeed, the bulk Fermi surface participates in the magneto-transport in parallel with the Fermi arc states in our WTe_2, which is consistent with our observation on the quantum oscillations in Fig. 1e. The establishment a Weyl orbit should satisfy the prerequisite that Fermi arc must be topologically protected. Recently, several spectroscopic works and calculations have revealed the existence of topologically protected Fermi arc in another type-II Weyl semimetal $MoTe_2$.[9,10,12] Furthermore, the theoretical work definitely demonstrated the topological protection of Fermi arc state in the (001) surface of WTe_2,[8] although there have been no transport evidences or reports on the topology of Fermi arc in the type-II Weyl semimetals till

now. Actually, the extra Weyl orbit quantum oscillation in our transport work is indirect evidence that Fermi arc is topologically protected. Besides the indirect observations in this work, we will provide three evidences to support the topological protection of Fermi arcs even if the bulk Fermi surfaces coexist with surface arc states.

Firstly, we have been also interested in the exploration of more evidences to reflect the nature of topologically protected Fermi arc state. We have investigated the spin orbit torques in WTe_2/ferromagnet bilayers at low temperatures. In this just finished but unpublished work, we found a much larger field-like spin orbit torque ($\tau_{\square}$) at low temperatures when current is applied along *b*-axis of WTe_2, compared to the result with *I*//*a*. This observation in WTe_2 multilayers strongly indicates that the topologically protected Fermi arc states ($\Gamma - Y$, *b*-axis) generate a large spin polarization when *I*//*b,* via spin-momentum locking. We give this data (Figure RS1) and detailed analysis in supplementary information for reviewers only.

Several works on the transport properties of the intensively studied topological insulators (TIs) have demonstrated the topological protection of the surface states, i.e. Aharonov-Bohm effect[14,15] in TIs nanoribbons and spin-momentum locking[16-18] in TIs/ferromagnet bilayers, although the Fermi level is deeply within the conduction band with a typical carrier density of $n \approx 10^{19} \sim 10^{21}\ \mathrm{cm}^{-3}$.[14-18] In those TIs, the bulk Fermi surface pockets always coexist with topological surface states. However, the topological surface states is not scattered into bulk. The carrier density in those TIs, which is related to the volume of bulk Fermi pockets, are even larger than that in WTe_2 ($n \approx 10^{19}\ \mathrm{cm}^{-3}$) in this study. We also tried to observe Aharonov-Bohm effect in our nanoribbons, but it turned out failed due to the fact that surface arc states are only present in (001) surfaces instead of (100) or (010) surfaces. This also agrees well with the theoretical calculation.[8] This can be considered as the second evidence that the surface state is protected against scattering even if the Fermi level is not within the gap.

Theoretically, the density of states should be near zero at the band crossing in Dirac semimetals and type-I Weyl semimetals. Practically, Dirac semimetal Cd_3As_2 (Ref. 12),[2] in which the extra Weyl orbit quantum oscillations were detected, has a moderate bulk Fermi pocket ($k_F = 3.3\times10^8\ \mathrm{m}^{-1}$, $n = 2.5\times10^{18}\ \mathrm{cm}^{-3}$).[2] It was found that the bulk Fermi surface pockets coexists with the topologically protected Fermi arc states as well. In the work on Cd_3As_2,[2] both bulk Fermi pocket and Weyl orbit quantum oscillation contribute simultaneously to two adjacent peaks in quantum oscillation FFT spectrum, which is rather similar to our observation in WTe_2. This can be considered as the third evidence.

The current manuscript bases their quantum oscillations analysis on Refs. 12 and 19, both of them written before the discovery of type II Weyl semimetals. In fact, the 'Weyl orbit' which produces the oscillations which involve the Fermi arcs should be modified or doesn't even exist in a type II WSM. For this reason I find the QO discussion confusing and I am not convinced that the data really supports the type II WSM hypothesis.

Reply: As the reviewer remind, the Weyl quantum oscillation is theoretically predicted before the proposal of type-II Weyl semimetals. The Weyl orbit quantum oscillation is a semi-classic analysis based on the existence of topologically protected surface state, Fermi arcs. We will briefly review the derivation and establishment of Weyl orbit quantum oscillation as proposed in Ref. 19.

As shown in Fig. 1a, four pairs of predicted Weyl nodes in WTe_2 exist in the (001) surfaces and each pair are separated by k_0 along the k_y direction.[8,11] If the electric field is applied along the k_y (or *b*) direction and the magnetic field is along z (or *c*) direction being normal to the slab, a Weyl orbit will be formed as following. When an electron is sliding through the Fermi arc on the top surface (or bottom one) of ribbon from the Weyl node "+" towards "-" Weyl node, it will

experience the Lorentz force $\partial_t \hbar \mathbf{k} = -e\mathbf{v}_\mathbf{k} \times \mathbf{B} = evB\hat{\mathbf{t}}_\mathbf{k}$. Here $\hat{\mathbf{t}}_\mathbf{k}$ is the unit tangent vector to the arc, and v the velocity. This Lorentz force leads to the electron shift along $|x|$ in real space. The time sliding along Fermi arc is $t_{arc} \approx k_0 / evB$. Once the electron approaches near "-" (or "+") bulk Weyl node, the chiral bulk LLs will transport the electron along *z* direction between the Fermi arcs of both surfaces, where the time propagating between top and bottom surface is $t_{bulk} \approx L / v$. Therefore, a complete Weyl orbit includes two surface Fermi arcs and two paths across the bulk LLs (Fig. 1b), which corresponds to a trajectory in xz plane of real space (upper panel of Fig. 1b). The total time completing a Weyl orbit is $t = 2(L + \frac{k_0}{eB}) / v$. According to the semi-classic quantum orbit, the energy levels should satisfy $E_n t = 2\pi(n+\gamma)$. It is easy to result to the conclusion of Ref. 19,

$$\frac{1}{B_n} = \frac{e}{k_0}\left(\frac{\pi v_F}{E_F}(n+\gamma) - L\right). \quad \text{(R1)}$$

From above review of theory that is proposed in Ref. 19, this analysis is suitable for all the Dirac semimetals and Weyl semimetals only if the topological surface states indeed exist. There are several hallmarks for a topological Weyl semimetal, including the direct observation of band structures and surface Fermi arcs[9,10], a negative longitudinal magnetoresistance related to the chiral anomaly[19], a new Fermi arc quantum oscillation[1], a nonlocal transport due to the chiral anomaly[20], and Klein tunneling[21], etc. In all the recent experimental demonstrations on Weyl semimetals, nobody can show the evidences of all the hallmarks of a Weyl point. Practically it is usually very difficult to demonstrate all. Therefore, two simultaneous observations in the same nanoribbon samples, extra Weyl orbit quantum oscillation and anisotropic chiral anomaly induced negative magnetoresistance, strongly support the existence of type-II Weyl semimetal in WTe_2.

Moreover, Ref. 19 predicts a thickness-dependent quantum oscillations frequency and the authors do not check for thickness.

Reply: We should consider two factors in the thickness-dependent quantum oscillation frequency, the phase offset of the peak versus 1/*B* and amplitude of Weyl orbit.

According to the theoretical prediction of Ref. 19, the dependence of peak position (B^{-1}) of Weyl orbit on thickness (*L*) is decided by Eq. (R1). First, the oscillation phase offset of the peaks (B^{-1}) in the curve ($d^2R/dB^2 - B^{-1}$) is expected to be thickness dependent although the oscillation period $1/\Delta B$ is independent on thickness. But it is really difficult to identify and extract the precise position (B^{-1}) of Weyl orbit due to the strong beating of Weyl orbit and bulk Fermi surfaces in SdH oscillations, because the peaks are close to each other in FFT spectra. Even though we can obtain the position of Weyl's *n*th Landau levels from the inverse FFT spectrum, the large uncertainty of the position will further hinder us from verifying Equation R1, the dependence of oscillation phase shift on the thickness. This similar case occurs in another experimental demonstration, Moll's work.[2]

Nevertheless, we can expect another kind of oscillation phase shift in the asymmetric Weyl orbit peak in FFT spectrum, arising from the non-adiabatic correction related to the field-induced tunneling between Fermi-arc states and bulk states. The shift of peak position (B^{-1}) towards higher fields will lead to a small hump towards low frequency in the FFT spectrum, which is expected for the non-adiabatic correction.[2] The asymmetric feature of Weyl orbit in our samples is shown in Fig. R2-1. The observation on asymmetric feature and non-adiabatic corrections are consistent with the work of Moll et al.[2]

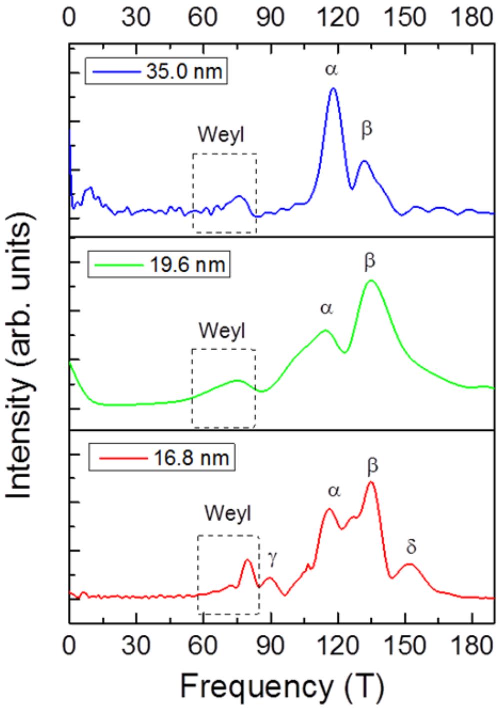

Figure R2-1 Asymmetric Weyl orbit peak in FFT spectra of nanoribbons with different thicknesses. The peaks in the dashed rectangles show asymmetric broadening towards low frequency due to the non-adiabatic correction.

On the other hand, the amplitude of Weyl orbit will decay with the increase of thickness. Ref. 19 predicts a ratio of surface to bulk amplitudes as[1]

$$\frac{\nu_{arc}}{\nu_{bulk}} \approx \frac{k_0}{k_F^2 L}, \qquad \text{(R2)}$$

where k_0, k_F and L are respectively Fermi arc length, Fermi radius and thickness. We added more newly measured data into Figure 4c to fit the thickness dependence of Weyl orbit amplitude. Figure R2-2 gives two best fits to the thickness dependence on the amplitude of Weyl orbit. The best fit to the data of decay of amplitude of Weyl orbit by Eq. (R2) will produce a coefficient $k_0 / k_F^2 - 7.04$ nm, which deviates heavily from the calculated value 0.22 nm with

$k_0 = 0.32\ \mathrm{nm}^{-1}$ and $k_F = 1.21\ \mathrm{nm}^{-1}$. If exponential behavior (e^{-L/L_0}) is taken into account to describe the thickness dependence, which is the same as Ref. 12, we will obtain $L_0 \approx 16.0\ \mathrm{nm}$ that is in the same magnitude of the mean free path $\approx 48.0\ \mathrm{nm}$.

We added the newly measured data and fitting into Fig. 4, as well as the discussion. Please see line 7 page 16 in main text.

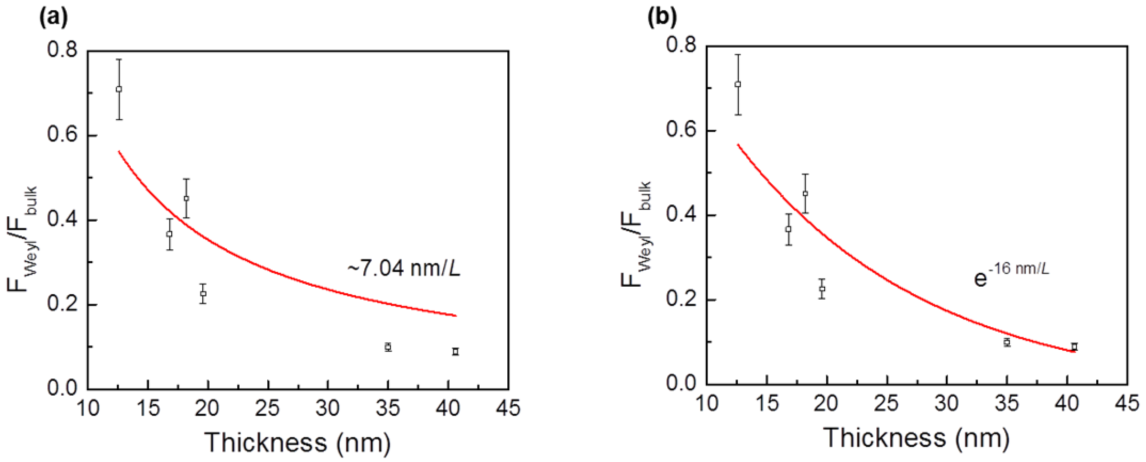

Fig. R2-2 Thickness dependent normalized Weyl orbit amplitude from FFT and its fitting. (a) Fitting by Eq. (R2), (b) exponential fitting by Ref. 12.

*I suggest to the authors to analyze the unique signatures of *bulk* quantum oscillations as proposed by O'Brien et al. in PRL 116, 236401. They propose that magnetic breakdown appears very distinctly.*

<u>Reply:</u> Thank you for your suggestion. Klein tunneling, which is proposed theoretically in PRL 116, 236401 , will induce an additional QO frequency $|F_+ - F_-|$ of two touching Fermi pockets, if the direction of magnetic field is applied perpendicular to the extremal area of Fermi pockets and Fermi level is near the position of potential Weyl points. As discussed in the literatures, this effect is very sensitive to the position of the chemical potential. In single crystal samples of WTe_2, both the first principle calculations and ARPES data indicate that the type II Weyl points

are located about 50~80 meV above the chemical potential. Therefore we are not very optimistic to see the Klein tunneling in this particular sample. As shown in Fig. R2-3a, in bulk WTe_2 two hole pockets (γ, δ, light blue) and two electron pockets (α, β, purple) are present in the Brillouin zone[22]. We should note that one smaller electron pocket α (and one smaller hole pocket γ) locates inside another larger electron pocket β (and another larger hole pocket δ), which is similar to a set of Russia dolls.[22] The potential Klein tunneling should occur between the larger electron (β) and larger hole (δ) pockets. According to our experimental 3D mapping of Fermi pockets of WTe_2(Fig. S5), the extremal area of Fermi surface in WTe_2 is the *bc* plane. We apply the magnetic field again, therefore, along *a*-axis to check the QO frequency very carefully. As shown in Fig. R2-3b, we found that location of four Fermi pockets is consistent with previous result in Fig. S5. Therefore, the potential additional frequency should be $\left|F_h - F_e\right|$ $= 363(\delta) - 330(\beta) \approx 33\,\mathrm{T}$. However, the data at low frequencies (0~100 T) is too noisy to identify the quantum oscillation due to Klein tunneling. This potential QO frequency $|F_+ - F_-|$ is neither evident nor can be excluded in our experiments. Hence we can't make a clear conclusion based on our observations. Therefore the fact that there is no obvious quantum oscillation at frequency $|F_+ - F_-|$ in our sample is consistent with the previous results from the ARPES measurements and DFT calculations.

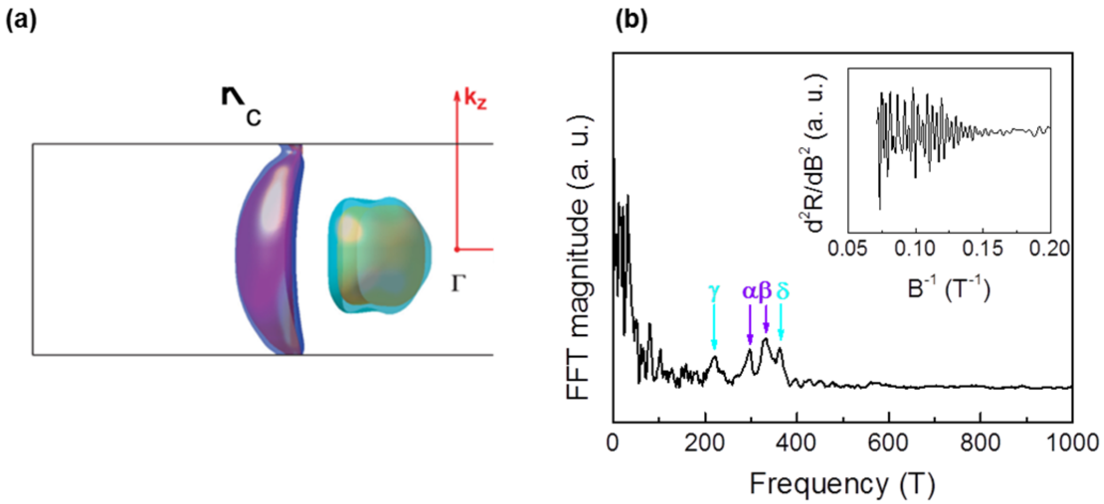

Fig. R2-3 (a) The bulk Fermi surfaces of WTe_2 from previous work[22]. The light blue surface is hole pocket, and purple surface is electron pocket. (b) The FFT spectrum of bulk WTe_2 with the magnetic field along *a*-axis of WTe_2. The inset give the SdH oscillation at 2 K.

According to another suggestion of this work (PRL 116, 236401), another powerful tool is to measure the de Haas-van Alphen effect in the magnetic susceptibility in the sample with type-II Weyl cones near the Fermi level. Although the Weyl points is near the Fermi level in our mechanically exfoliated several-layered WTe_2, the amount of several-layered samples is, however, far insufficient to detect the de Haas-van Alphen effect in the magnetic susceptibility. Therefore, fabrication of large amount of the single crystal phase of electron-doped bulk WTe_2 is an obstacle for us at this stage if we want to check the Klein tunneling through de Haas-van Alphen effect.

We have added the discussion about Klein tunneling into SI note 2.

Reference

1 Potter, A. C., Kimchi, I. & Vishwanath, A. Quantum oscillations from surface Fermi arcs in Weyl and Dirac semimetals. *Nat Commun* **5**, 5161 (2014).

2 Moll, P. J. *et al.* Transport evidence for Fermi-arc-mediated chirality transfer in the Dirac semimetal Cd3As2. *Nature* **535**, 266 (2016).

3 Dos Reis, R. *et al.* On the search for the chiral anomaly in Weyl semimetals: The negative longitudinal magnetoresistance. *New J Phys* **18**, 085006 (2016).

4 Arnold, F. *et al.* Negative magnetoresistance without well-defined chirality in the Weyl semimetal TaP. *Nat Commun* **7**, 11615 (2016).

5 Thornton, T., Roukes, M., Scherer, A. & Van de Gaag, B. Boundary scattering in quantum wires. *Phys Rev Lett* **63**, 2128 (1989).

6 De Jong, M. & Molenkamp, L. Hydrodynamic electron flow in high-mobility wires. *Phys Rev B* **51**, 13389 (1995).

7 Tang, S. *et al.* Quantum spin Hall state in monolayer 1T'-WTe2. *Nat Phys* **13**, 683 (2017).

8 Soluyanov, A. A. *et al.* Type-II Weyl semimetals. *Nature* **527**, 495-498 (2015).

9 Deng, K. *et al.* Experimental observation of topological Fermi arcs in type-II Weyl semimetal MoTe2. *Nat Phys* **12**, 1105 (2016).

10 Huang, L. *et al.* Spectroscopic evidence for a type II Weyl semimetallic state in MoTe2. *Nat Mater* **15**, 1155 (2016).

11 Wang, C. *et al.* Observation of Fermi arc and its connection with bulk states in the candidate type-II Weyl semimetal WTe 2. *Phys Rev B* **94**, 241119 (2016).

12 Jiang, J. *et al.* Signature of type-II Weyl semimetal phase in MoTe2. *Nat Commun* **8**, 13973 (2017).

13 Neupane, M. *et al.* Observation of a three-dimensional topological Dirac semimetal phase in high-mobility Cd3As2. *Nat Commun* **5**, 3786 (2014).

14 Peng, H. *et al.* Aharonov-Bohm interference in topological insulator nanoribbons. *Nat Mater* **9**, 225-229 (2010).

15 Xiu, F. *et al.* Manipulating surface states in topological insulator nanoribbons. *Nat Nanotechnol* **6**, 216-221 (2011).

16 Ando, Y. *et al.* Electrical detection of the spin polarization due to charge flow in the surface state of the topological insulator Bi1. 5Sb0. 5Te1. 7Se1. 3. *Nano Lett* **14**, 6226-6230 (2014).

17 Li, C. *et al.* Electrical detection of charge-current-induced spin polarization due to spin-momentum locking in Bi2Se3. *Nat Nanotechnol* **9**, 218-224 (2014).

18 Tang, J. *et al.* Electrical detection of spin-polarized surface states conduction in (Bi0. 53Sb0. 47) 2Te3 topological insulator. *Nano Lett* **14**, 5423-5429 (2014).

19 Nielsen, H. B. & Ninomiya, M. The Adler-Bell-Jackiw anomaly and Weyl fermions in a crystal. *Phys Lett B* **130**, 389-396 (1983).

20 Parameswaran, S., Grover, T., Abanin, D., Pesin, D. & Vishwanath, A. Probing the chiral anomaly with nonlocal transport in three-dimensional topological semimetals. *Phys Rev X* **4**, 031035 (2014).

21 O'Brien, T., Diez, M. & Beenakker, C. Magnetic breakdown and Klein tunneling in a type-II Weyl semimetal. *Phys Rev Lett* **116**, 236401 (2016).

22 Zhu, Z. *et al.* Quantum oscillations, thermoelectric coefficients, and the fermi surface of semimetallic WTe 2. *Phys Rev Lett* **114**, 176601 (2015).

Reviewers' comments:

Reviewer #1 (Remarks to the Author):

Dear Editors,

I am glad my comments received attention by the authors of this manuscript. The new version including the supplementary information has answered all of my comments and questions satisfactorily. Some of the technical things such as error bars and transport setup details were added. Especially the careful considerations of the current jetting and Knudsen effects are necessary to support the evidence for the negative MR originating from a chiral anomaly.
In addition, the improved manuscript now contains a proper investigation of the thickness dependence.
I now have only minor comments left, see the table below.

Line Comment
17 I am not sure if one should call Weyl orbit a technique

299 Predicated ◊ predicted

301 It sounds as if you somehow relate your 78 T frequency with that effect. Clearly it would be a much smaller F since it is the difference |F+-F-|
Question: How would the probability for Klein tunneling depend on thickness (i.e. the shift in chemical potential)? Could the exponential decrease of the oscillation amplitude be explained by such an effect, too?

[REDACTED]

With this, I would like to keep my response short. I would definitely recommend this piece of work for publication in Nature Communication in its current state.

Best regards,

Reviewer #2 (Remarks to the Author):

The authors have made several points clearer in the current version of the manuscript. However, I am still confused about their main claim that WTe_2 is a type II Weyl semimetal. In the beginning of the paper they claim that in order to show that a sample is indeed a typeII WSM it should be established that there is negative magnetoresistence due to the chiral anomaly and surface quantum oscillations in addition to the bulk ones.
I have two problems with this:

1. These claims do not distinguish between typeI and typeII WSMs. Both the chiral anomaly and the surface Fermi arcs are expected to occur in typeI WSMs. The authors cite five papers supporting their criterion (Refs. 12, 19, 4, 7 and 20) where none of them actually discuss typeII WSMs in particular. The theory papers are written for a typeI WSM while the experiments do not define any criterion.

2. The authors have found more than one oscillations frequency. However, in my opinion they do not show enough convincing information to support the claim that the lowest of these oscillations is indeed related to the surface. The only exception is the fact that the amplitude of Fs almost

vanishes when the sample is thick.

In fact, some detailed calculations suggest that in typeII WSMs the chiral Landau level is absent and therefore the surface quantum oscillations can not form.

To summarize, the authors show evidence supporting the claim that WTe_2 is a Weyl semi-metal but are not in position to determine whether it is typeI or typeII

Reply to the comments of NCOMMS-17-13980A

Response to Reviewer #1 :

Reviewer #1 (Remarks to the Author): I am glad my comments received attention by the authors of this manuscript. The new version including the supplementary information has answered all of my comments and questions satisfactorily. Some of the technical things such as error bars and transport setup details were added. Especially the careful considerations of the current jetting and Knudsen effects are necessary to support the evidence for the negative MR originating from a chiral anomaly. In addition, the improved manuscript now contains a proper investigation of the thickness dependence.

Reply: Thank you for your very positive comments.

I now have only minor comments left, see the table below.

Line Comments:

17 I am not sure if one should call Weyl orbit a technique

Reply: We delete this ambiguous expression.

299 Predicated □ predicted

Reply: Corrected.

301 It sounds as if you somehow relate your 78 T frequency with that effect. Clearly it would be a much smaller F since it is the difference |F+-F-|

Question: How would the probability for Klein tunneling depend on thickness (i.e. the shift in chemical potential)? Could the exponential decrease of the oscillation amplitude be explained by such an effect, too?

Reply: Yes, the potential frequency caused by Klein tunneling, $|F_+-F_-|$, is much smaller than the frequency that we observed near 78 T.

Although the theory proposed by O'Brien et al on a type-II Weyl semimetal did not discuss the thickness effect in real space on Klein tunneling, we can still expect that the oscillation amplitude related to Klein tunneling should increase with the decrease of nanoribbon thickness if we can detect the Klein tunneling. This is because Klein tunneling requires that the Fermi level must be near the location of Weyl points. According to our own data in Fig. 1 and Fig. S3, the chemical potential in WTe_2 nanoribbons indeed increased and approached the position of Weyl points as the thickness decreased. However, Klein tunneling requires the experimental configuration in which the applied magnetic field is perpendicular to the extremal area of Fermi surface, i.e. *B*//*a* (the extremal area of Fermi surface in WTe_2 is the k_y-k_z plane, Fig. S5). This magnetic field setup is different from that in Weyl orbit measurement (*B*//*c*). When we applied magnetic field along *a*-axis of WTe_2 nanoribbons (t<40 nm), we cannot observe any oscillatory behavior for *B*//*a* (See Fig. 3a). Therefore, the exponential decrease of the oscillation amplitude (78 T frequency) in Fig. 4 should not be related to such an effect.

The additional data on spin-orbit torque shared with the reviewers is impressive and provides strong support for the proclaimed Weyl physics in WTe_2.

With this, I would like to keep my response short. I would definitely recommend this piece of work for publication in Nature Communication in its current state.

Reply: Thank you very much for your highly positive comment on our work.

Response to Reviewer #2 :

Reviewer #2:

The authors have made several points clearer in the current version of the manuscript. However, I am still confused about their main claim that WTe2 is a type II Weyl semimetal. In the beginning of the paper they claim that in order to show that a sample is indeed a typeII WSM it should be established that there is negative magnetoresistence due to the chiral anomaly and surface quantum oscillations in addition to the bulk ones.

I have two problems with this:

1. These claims do not distinguish between typeI and typeII WSMs. Both the chiral anomaly and the surface Fermi arcs are expected to occur in typeI WSMs. The authors cite five papers supporting their criterion (Refs. 12, 19, 4, 7 and 20) where none of them actually discuss typeII WSMs in particular. The theory papers are written for a typeI WSM while the experiments do not define any criterion.

Reply: Thanks for your further comments that encouraged us to improve our manuscript further, particularly on this critical issue.

Indeed, all the transport works we cited are referring to the features of type-I Weyl fermions, because there have been no reports on the definition of the transport criteria for type-II Weyl fermions so far. According to the definition of a type-II Weyl semimetal, a quasiparticle can be regarded as a type-II Weyl fermion that has the following characteristics. Along the *k* direction that Weyl cone tilts towards, its kinetic energy is larger than its potential one in low-energy condensed matter physics.[1] More specially, type-II Weyl fermions violate the Lorentz symmetry strongly.[1,2] Recently, it was reported that the decisive criterion for a type-II Weyl semimetal is the direct observation of its tilted band crossing in three directions in the momentum space, as demonstrated in LaAlGe (Figure R1).[2] However, it is impossible to use the same technique to confirm that WTe_2 is a type-II Weyl semimetal, because the separation between two Weyl points

in WTe_2 is too small and beyond the resolution of ARPES, although several groups attempted to do so.[3-6]

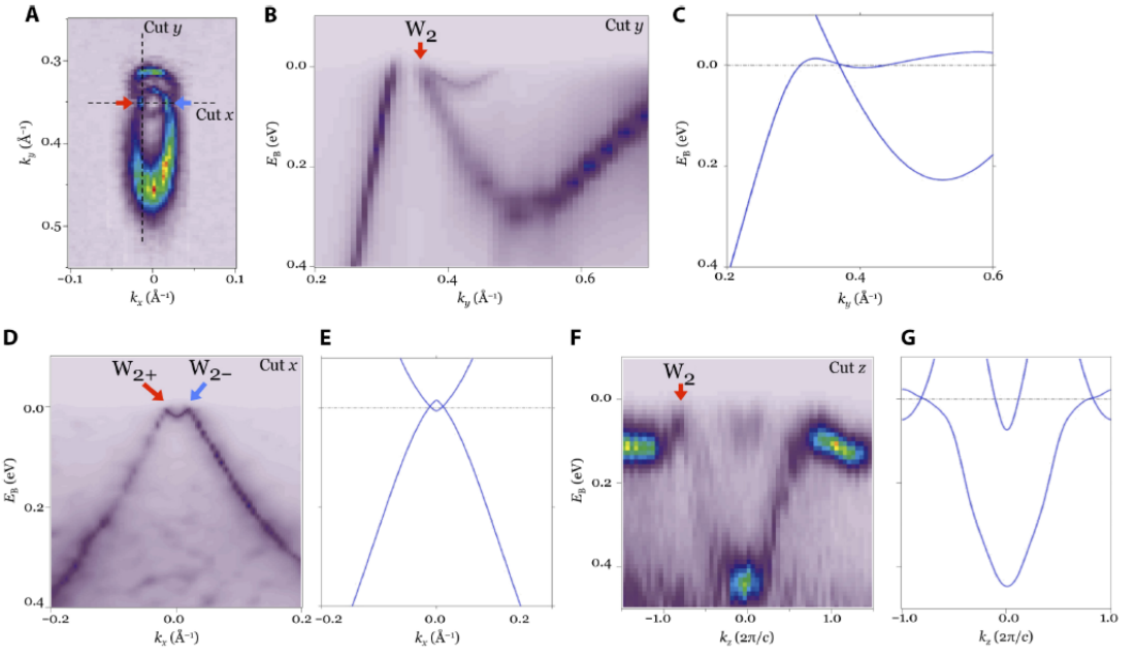

Figure R1 The decisive criterion on the determination of type-II Weyl semimetal, i.e. LaAlGe.[2] This Figure comes from Ref. 2. The energy spectra should be detected in three directions. (B) and (C) give the measured and calculated spectra along k_y; (D) and (E) for k_x; (F) and (G) for k_z.

In order to explore the type of Weyl fermions in WTe_2, we have no choice but integrating the calculation and transport evidence. Besides several band structure calculations,[1,4,7] it has been established that the anisotropy of chiral anomaly should be a strong evidence to distinguish the type-II Weyl semimetals from type-I Weyl semimetals, in addition of the direct observation of the tilted bulk Weyl cones using ARPES[1,8]. In Type-I Weyl semimetals, the chiral anomaly always appears regardless of the direction of the applied magnetic fields.[8] However, in type-II Weyl semimetals, the chiral Landau levels and chiral anomaly exists only when the magnetic field is applied in the direction along which the kinetic energy of the fermions is larger than their potential energy[1,4,9-11]. Specifically, the ratio of the kinetic energy (T_k) to potential energy (U_k),

$R = T^2(k)/U^2(k) > 1$ was proposed to be a criterion to identify the directions along which a Weyl fermion is type-II and has the chiral anomaly[1]. This phenomenon should be a direct evidence for type-II Weyl semimetals. In WTe_2, the magnetic field must be applied along *b*–axis.[1,4] When the magnetic field is applied normal to the tilt direction of the Weyl fermion cones in a type-II Weyl semimetal, the chiral Landau levels are absent[9-11] and thus chiral anomaly (the longitudinal negative magnetoresistance) will disappear[1]. This is what we observed in our experiments.

Weyl orbit is another indirect evidence for the existence of Weyl points, although it can't provide the information about the type of the Weyl semimetals. Following the critical comment raised by the reviewer, the Weyl orbit in type-II Weyl semimetals should be slightly modified from the previously proposed physical model based on type-I Dirac and Weyl semimetals, whereas the main conclusions on the Weyl orbit, i.e. oscillation frequency and 2D feature, keep unchanged. Please see the detailed information in reply to comment 3.

Therefore, based on our two observations, the direct evidence (anisotropy of chiral anomaly) and the indirect evidence (Weyl orbit), WTe_2 should be regarded as a type-II Weyl semimetal. We have modified the introduction part in the revised manuscript accordingly, especially on the criterion to distinguish type-I and type-II Weyl semimetals. Please see line 18 on page 2, lines 7, 12 on page 3, line 2 on page 4.

Comment 2. The authors have found more than one oscillations frequency. However, in my opinion they do not show enough convincing information to support the claim that the lowest of these oscillations is indeed related to the surface. The only exception is the fact that the amplitude of Fs almost vanishes when the sample is thick.

Reply: In principle, at most only four frequencies should be observed on a bulk WTe_2 sample, because four bulk Fermi surfaces (two electron and two hole pockets) will contribute to the SdH oscillations. In order to confirm that the extra peak comes from Weyl orbit, we mapped the Fermi surfaces of bulk WTe_2 (Fig. S5), the four peaks agree well with both the oscillations

observed experimentally in the measurements of Seebeck effect[12] and the results obtained by band structure calculations.[1,4,7]

Interestingly, we observed the fifth peak near ~78 T that cannot be accounted for by the bulk property. We then attributed this peak to the formation of the "Weyl orbit". The systematic study on the extra peak reveals that the amplitude of this Weyl orbit frequency not only depends on the sample thickness, but also follows a $\cos^{-1}\theta$ angular dependence (Fig. 2 in main manuscript), a unique property of a two-dimensional orbit and a strong evidence of 2D characteristic of Weyl orbit.[13,14] Furthermore, the frequency near ~78 T is consistent well with the Weyl orbit frequency calculated with the Fermi arc length, but it differs completely from the characteristic frequency of the potential Klein tunneling $|F_h - F_e| = 363(\delta) - 330(\beta) \approx 33\,\mathrm{T}$.[15] Therefore, the evidences presented here should be strong enough to demonstrate that the origin of the extra peak is due to the Weyl orbit. We have modified model of Weyl orbit in type-II Weyl semimetals motivated by your suggestions, the details will be shown below.

Comment 3. In fact, some detailed calculations suggest that in type-II WSMs the chiral Landau level is absent and therefore the surface quantum oscillations cannot form.

Reply: Thank you for your critical comment to encourage us to think more on the formation of Weyl orbit in type-II Weyl semimetals. Based on your critical comment, we have modified the Weyl orbit as shown below.

According to several detailed calculations on the Landau levels in type-II Weyl semimetals,[9-11] the chiral Landau levels are still visible (Figure R2(h)[10] and Figure R3A) when the magnetic field is applied along the tilting direction of Weyl cone. This is why we still can observe the chiral anomaly induced longitudinal negative magnetoresistance in *b*-axis WTe_2 nanoribbons (Fig. 3b&3c).

When the magnetic field is normal to the tilting direction of type-II Weyl cone, $B(z) \perp k_{\text{Weyl tilt}}(x)$, the chiral Landau levels are missing indeed, as shown in Figure R2(g).[10] Therefore, as pointed out by the reviewer, the missing of chiral Landau level states will indeed lead to a small modification in the formation of Weyl orbit in type-II Weyl semimetals, but the main conclusion of Weyl orbit proposed by A.C. Potter will remain unchanged. We will describe the formation of Weyl orbit in type-II Weyl semimetals in the following.

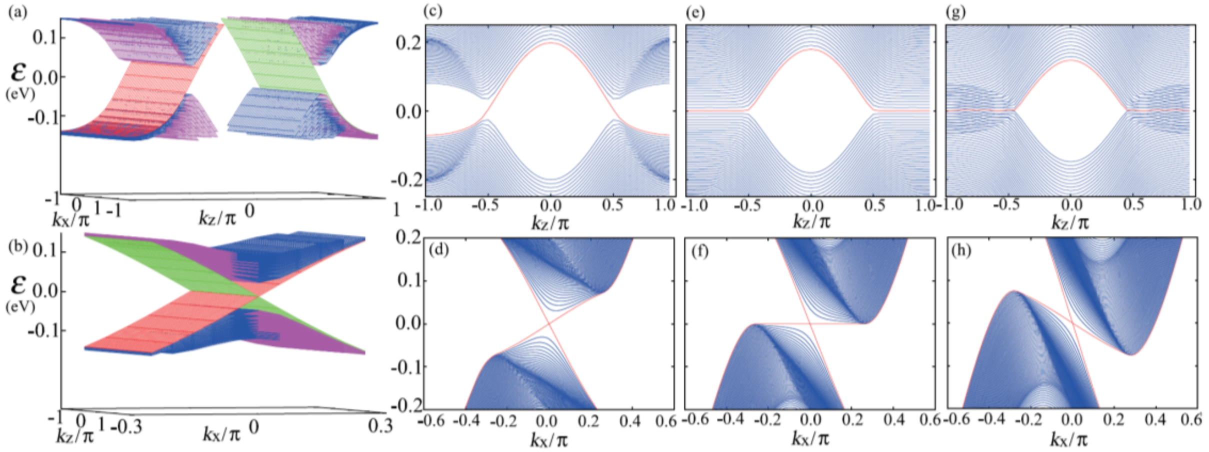

FIG. 2. Energy spectra are obtained for $L_y = 400$, for (a), (c), (e), and (g): $\mathbf{B}\|\mathbf{z}$ and (b), (d), (f), (h): $\mathbf{B}\|\mathbf{x}$. The values of t_{0x} are chosen

Figure R2 The calculated Landau levels of type-II Weyl semimetals by Serguei et al.[10] The separation direction of Weyl point is along *z* $k_{\text{Weyl}} = k_z = (0, 0, \pm\pi/2)$, but the Weyl cone tilts along *x* direction. Figures (a), (c), (e), (g) corresponds to the calculated Landau levels for B//z, and (b), (d), (f), (h) corresponds to these for *B*//*x*. (c) and (d) refer to the Landau levels of type-I Weyl semimetal, and (g) and (h) refer to that of type-II Weyl semimetal.

First, we calculated the Landau levels in type-II Weyl semimetals for the exact same configurations as that in our measured WTe_2: Weyl cone tilting along $\Gamma - Y$,[1,4] Weyl points separating along $\Gamma - Y$, the magnetic field applied along *b*- (*B*//*b*, Fig. R3A) and *c*-axis (*B*//*c*, Fig. R3B). Figure R3 shows the Landau levels with different magnetic field configuration. When the applied magnetic field is parallel to *b*-axis, i.e. *B*//*b*, we found that the chiral Landau levels are still well-defined (Fig. R3A), consistent with the result in Fig. 2(h). This is why we can still

observe the chiral anomaly induced negative longitudinal magnetoresistance in *b*-axis WTe_2 nanoribbons (Figs. 3b&3c in main text). Whereas, when the applied magnetic field is normal to the tilting direction of Weyl cones the chiral Landau levels are missing (Fig. R3B), which agrees well with Fig. 2(g) as well. Note that our calculation is a little different from the calculation shown in Figure R2(g), because the tilting direction of Weyl Cones ($\Gamma - \mathrm{X}$) is set differently from the separation direction of Weyl points ($\Gamma - \mathrm{Z}$) in Serguei's calculation.[10] In Dirac and type-I Weyl semimetals, the chiral Landau levels ($n = 0$) act as the only one-way "conveyor-belt", because the bulk Landau levels near the Fermi level in Dirac and type-I Weyl semimetals are gapped.[13] However, in type-II Weyl semimetals, when the magnetic field is normal to the tilting direction of Weyl cone, i.e. in WTe_2 *B*//*c*, the electron and hole pockets are not gapped and there are still several bulk Landau level states ($n \geq 1$) with opposite Fermi velocity near the Fermi level (Fig. R3B). Therefore, in a type-II Weyl semimetal, when the electrons arrive the Weyl points through topological Fermi arc states, they can traverse the nanoribbon via bulk Landau levels ($n \geq 1$) near the Weyl points. The bulk Landau levels ($n \geq 1$) with opposite Fermi velocities (up and down) can act as the paths to transport the electrons between the top and bottom surfaces (Fig. R3C). Therefore, the Weyl orbit forms in type-II Weyl semimetals, in which two Fermi arcs and two bulk Landau levels ($n \geq 1$) with opposite Fermi velocities are involved.

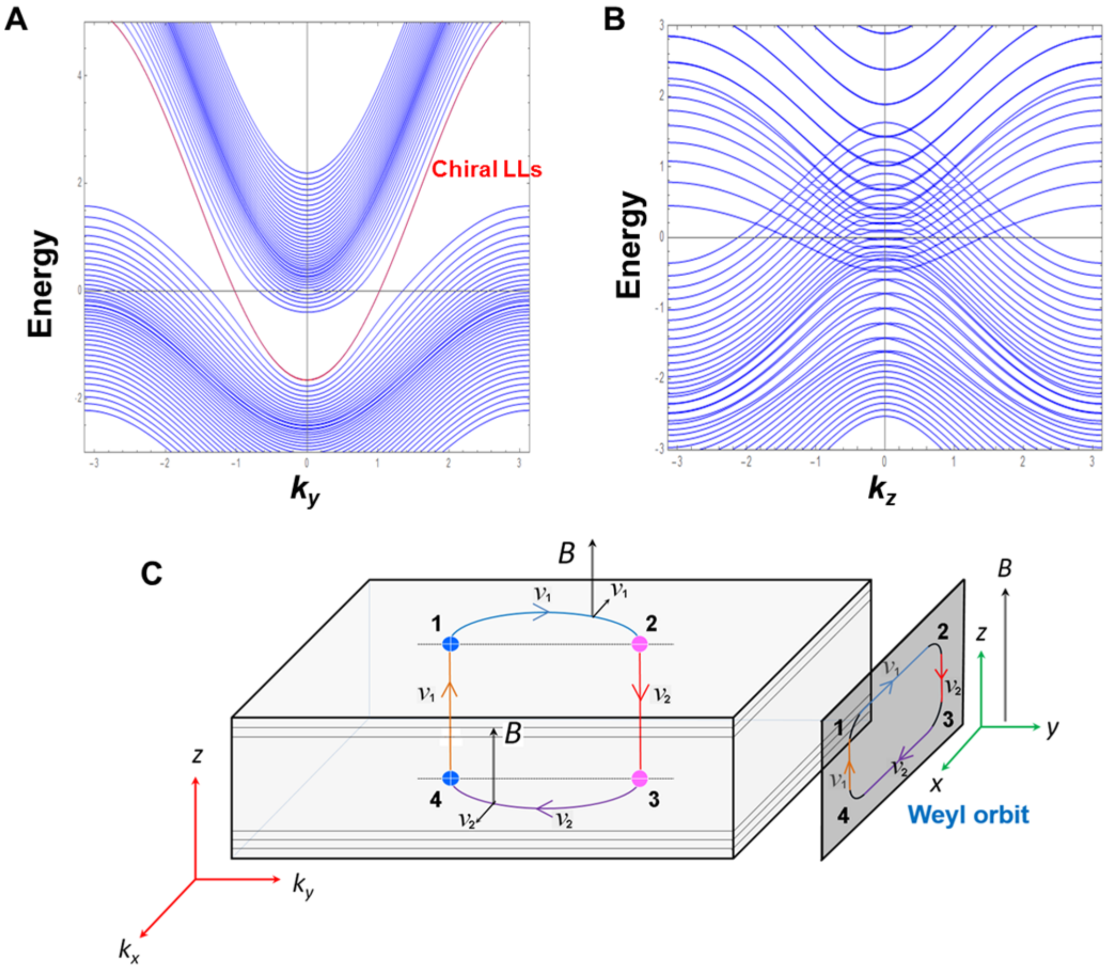

Figure R3 Modified Weyl orbit in type-II Weyl semimetals. The calculated Landau levels in a type-II Weyl semimetal, where the Weyl cone tilts along $\Gamma - \mathrm{Y}$ direction. (**A**) A magnetic field is applied parallel to the tilting direction of Weyl cones ($\Gamma - \mathrm{Y}$), i.e. *B*//*b*; (**B**) A magnetic field is applied normal to the tilting direction of Weyl cone, i.e. *B*//*c*. (**C**) The modified Weyl orbit in mixed real and momentum space. Weyl orbit are consisted of four paths, two Fermi arcs (12 and 34), two bulk Landau level states with opposite Fermi velocity (23 and 14). The side view give the electron trajectory in real space, which is the same as the model proposed by A.C. Potter.[13]

After the slight modification on the Weyl orbit, we should notice that there are a lot of bulk Landau level states ($n \geq 1$) near the Fermi level. One will naturally ask a question. Will the

scattering between the bulk Landau levels ($n \geq 1$) be too strong to destroy the Weyl orbit? To answer this question, we have carefully examined the time elapsed between the top surface and bottom surface. Fortunately, the Fermi velocity of the bulk electrons along *c* direction in our WTe_2 is as high as $\overline{v}_F = \hbar \overline{k}_c / \overline{m}^* \approx 3.09 \times 10^5\ \mathrm{m/s}$ (Fig. S4 and S5), the mean free path for the bulk electron is around $l = v_F \overline{\tau} \approx 48\ \mathrm{nm}$, if coherent scattering time of both electron pockets $\overline{\tau} \approx (1.6 \pm 0.1) \times 10^{-13}\ \mathrm{s}$ (Fig. S8) is considered. This mean free path is even longer than the thickness of the thickest sample (~35 nm) in which we could observe the Weyl orbit as shown in Fig. 4. Therefore, the electrons will finish the transport between two Fermi arcs before it was scattered off by other bulk Landau levels near Fermi level. Hence, when the magnetic field is applied normal to the tilting direction of Weyl cone (i.e. *B*//*c*), a Weyl orbit will be formed in a type-II Weyl semimetal only if the bulk Landau levels can substitute the missing chiral Landau levels.

Now, let's examine the time spent on each path and to find the Weyl orbit frequency. Figure R3C shows the Weyl orbit in a type-II Weyl semimetal that is composed of two Fermi arcs (momentum trajectories) and two bulk Landau levels (real space trajectories). The velocities of the bulk Landau levels (positive and negative Fermi velocity) in type-II Weyl semimetals should be slightly different due to the different slops of the energy spectra (Fig. R3B). We define the velocity on path 41 and 12 is v_1, and the velocity on path 23 and 34 is v_2. The time sliding along Fermi arc on top (bottom) surface is $t_{arc\ top} \approx k_0 / e v_1 B$ ($t_{arc\ bottom} \approx k_0 / e v_2 B$). The propagating time between top and bottom surface is $t_{bulk\ 23} \approx L / v_2$ ($t_{bulk 41} \approx L / v_1$, L is the thickness of nanoribbon). Therefore, The total time completing a Weyl orbit is $t = (v_1 + v_2)(L + \frac{k_0}{eB}) / v_1 v_2$. Given the average velocity along *c* is $\overline{v}_F = 2 v_1 v_2 / (v_1 + v_2)$ and the semi-classic quantum orbit

requirement on the energy $E_n t = 2\pi(n+\gamma)$, it is found that the result is the same as obtained in Ref. 19,

$$\frac{1}{B_n} = \frac{e}{k_0}\left(\frac{\pi\bar{v}_F}{E_F}(n+\gamma) - L\right). \tag{S3}$$

Therefore, we found that the key results in the Weyl orbit in type-II Weyl semimetals remain the same as that in type-I Weyl semimetals that was proposed by A.C. Potter, including the quantum oscillation frequency $F_S = \frac{E_F k_0}{e\pi\bar{v}_F}$, thickness dependent quantum oscillations frequency.

The modified Weyl orbit in type-II Weyl semimetals are discussed in main text and SI Please see page 4 line 2 and page 8 line 7 in main text, and note1 (page 18) in SI.

To summarize, the authors show evidence supporting the claim that WTe_2 is a Weyl semi-metal but are not in position to determine whether it is typeI or typeII.

Reply: Thanks for your partial positive comment on our conclusion. Following your critical comment, we made the modification in the formation of Weyl orbit in the type-II Weyl semimetals and discuss the decisive criterion to distinguish between type-I and type-II Weyl semimetals in introduction and above. We believe that our experimental observations, together with several band crossing calculations, strongly support the claim that WTe_2 is a type-II Weyl semimetal. We also hope that you are able to be convinced by our explanations.

Reference

1 Soluyanov, A. A. *et al.* Type-II Weyl semimetals. *Nature* **527**, 495-498 (2015).

2 Xu, S. Y. *et al.* Discovery of Lorentz-violating type II Weyl fermions in LaAlGe. *Sci Adv* **3**, doi:ARTN e1603266

10.1126/sciadv.1603266 (2017).

3 Bruno, F. Y. *et al.* Observation of large topologically trivial Fermi arcs in the candidate type-II Weyl semimetal WT e 2. *Phys Rev B* **94**, 121112 (2016).

4 Wang, C. *et al.* Observation of Fermi arc and its connection with bulk states in the candidate type-II Weyl semimetal WTe 2. *Phys Rev B* **94**, 241119 (2016).

5 Wu, Y. *et al.* Observation of Fermi arcs in the type-II Weyl semimetal candidate WTe 2. *Phys Rev B* **94**, 121113 (2016).

6 Belopolski, I. *et al.* Fermi arc electronic structure and Chern numbers in the type-II Weyl semimetal candidate MoxW1-xTe2. *Phys Rev B* **94**, doi:ARTN 085127

10.1103/PhysRevB.94.085127 (2016).

7 Chang, T. R. *et al.* Prediction of an arc-tunable Weyl Fermion metallic state in MoxW1-xTe2. *Nat Commun* **7**, doi:ARTN 10639

10.1038/ncomms10639 (2016).

8 Lv, Y.-Y. *et al.* Experimental Observation of Anisotropic Adler-Bell-Jackiw Anomaly in Type-II Weyl Semimetal WTe 1.98 Crystals at the Quasiclassical Regime. *Phys Rev Lett* **118**, 096603 (2017).

9 Tchoumakov, S., Civelli, M. & Goerbig, M. O. Magnetic-Field-Induced Relativistic Properties in Type-I and Type-II Weyl Semimetals. *Phys Rev Lett* **117**, 086402, doi:ARTN 086402

10.1103/PhysRevLett.117.086402 (2016).

10 Udagawa, M. & Bergholtz, E. J. Field-Selective Anomaly and Chiral Mode Reversal in Type-II Weyl Materials. *Phys Rev Lett* **117**, 086401 (2016).

11 Yu, Z. M., Yao, Y. G. & Yang, S. Y. A. Predicted Unusual Magnetoresponse in Type-II Weyl Semimetals. *Phys Rev Lett* **117**, 077202, doi:ARTN 077202

10.1103/PhysRevLett.117.077202 (2016).

12 Zhu, Z. *et al.* Quantum oscillations, thermoelectric coefficients, and the fermi surface of semimetallic WTe 2. *Phys Rev Lett* **114**, 176601 (2015).

13 Potter, A. C., Kimchi, I. & Vishwanath, A. Quantum oscillations from surface Fermi arcs in Weyl and Dirac semimetals. *Nat Commun* **5**, 5161 (2014).

14 Moll, P. J. *et al.* Transport evidence for Fermi-arc-mediated chirality transfer in the Dirac semimetal Cd3As2. *Nature* **535**, 266 (2016).

15 O'Brien, T., Diez, M. & Beenakker, C. Magnetic breakdown and Klein tunneling in a type-II Weyl semimetal. *Phys Rev Lett* **116**, 236401 (2016).
